# SF‐CORNER (splenic flexure colorectal cancer): an international survey of operative approaches and outcomes for cancers of the splenic flexure

**DOI:** 10.1111/codi.16895

**Published:** 2024-02-12

**Authors:** H. Sekhar, M. Dyer, M. Khan, P. J. Mitchell, N. P. West, S. Moug, D. Vimalachandran, the SF‐CORNER collaborative study group, A. Tidjane, N. Dudi‐Venkata, H. Mohan, T. Sammour, E. Samadov, G. Van Ramshorst, G.M. Gomes, G.A. Laporte, M. Slavchev, D. O’Reilly, I. Sallam, M. Shalaby, E. Duchalais, B. Seeliger, W. Hohenberger, U. Ronellenfitsch, F. Gyamfi, I. Katsoulis, K. Stamou, A. Mehuj, S. Rajan, J. Larkin, E. Ryan, E. Baldini, M. Campanell, G. Canonico, G. Capolupo, M. Caricato, F. Colombo, P. DeNardi, C. Feo, R. Galleano, P. Lapolla, G. Lisi, V. Lizzi, D. Matteo, S. Novello, F. Pata, T. Perra, E. Pinotti, M. Romano, F. Rosa, M. Rottoli, P. Sileri, A. Taddei, L. Tirloni, H. Ueno, F. Fa, A. Dulskas, N. Samalavicius, A. Souadka, M. Benitez, E. Divan, C. Soule, H. Kroon, A. Adeyeye, J.G. Makama, H.L. Thorsen, F. Grama, I. Negoi, A. Vardanyan, J. Escartin, M. Estaire‐Gómez, M.H. Garcia, F. Mendoza‐Moreno, M. Prats, M. Rutegard, S. Atici, E. Colak, M. Tanal, D. Keller, S. Wexner, N. Djelali, A. Hebbar, S. Mesli, Y. Arafat, N. Blefari, T. Chittleborough, Y.H. Lam, S. Stevens, E. van Eetvelde, A. Awad, M. Coura, C. da Silva Cardial, M. Ebrahim, H. Elfeki, D. Gill, A. Sakr, F.M. Vieira, A. Castaldi, J. Loriau, C. Niki, M. Pocard, N. Regent, J.J. Tuech, R. Awoonor‐Williams, M. Frountazas, N. Michalopoulos, D. Schizas, T. Sidiropoulos, M. Sotiropoulou, A. Syllaios, P. Vassiliu, R. Cahill, M. Flanagan, M. Kelly, N. Lynch, L. O’Connell, J. Ryan, G. Alemanno, M. Ardu, G. Cerino, G. Gallo, F. Ghignone, I. Iannone, A. Mingoli, I. Montroni, T. Nelli, G. Pellino, M.V. Papa, A. Porcu, D. Sasia, S. Di Saverio, N. Tamini, C. Tanda, P. Ziprin, M. Piccoli, D. Venskutonis, M. Thanapal, M. Trego‐Avila, M. Anass, M. Hamid, A. Lin, A. Adejumo, O. Adewunmi, O. Adeyemi, B. Aminu, I. Chukwu, I. Garzali, S. Irmiya, N.A. Khan, H.B. Hernandez, N. Figueiredo, E.A. Bonci, O. Gingina, S.T. Makkai‐Popa, A.M. Musina, D. Lutrin, S. Sifuba, J.M. Alegre, M. Alvarez‐Gallego, P. Baños, V. Carneros, C.J. Gómez‐Díaz, S. Jeri‐McFarlane, B. Matias, C. Placer‐Galán, P. Tejedor, V. Vigorita, K. Tsimogiannis, C. Akyol, O. Bozbiyik, O. Duzgun, E. Kamer, A. Isik, S. Leventoglu, N. Okkabaz, I. Ozata, V. Ozben, A. Ozcan, O. Ozkan, A. Şanli, E. Sivrikoz, H. Ulgur, P. Okeny, P. Barrow, A. Beggs, A. Bhowmick, S. Chapman, J. Davies, G. Faulkner, N. Heywood, A. Renwick, K. Sahnan, M.H. Siddique, C. Smart, P. Sutton, D. Watt, L.W. Wheldon, M. Whiteford

**Affiliations:** ^1^ Department of General and Colorectal Surgery The Newcastle upon Tyne Hospitals NHS Foundation Trust Newcastle upon Tyne UK; ^2^ Foundation trainee Swansea Bay University Health Board Swansea UK; ^3^ Department of General and Colorectal Surgery Blackpool Teaching Hospitals NHS Foundation Trust Blackpool UK; ^4^ Department of General and Colorectal Surgery Lancashire Teaching Hospitals NHS Foundation Trust Preston UK; ^5^ Faculty of Medicine and Health, School of Medicine University of Leeds Leeds UK; ^6^ Department of General and Colorectal Surgery Royal Alexandra Hospital Paisley, Glasgow UK; ^7^ Department of General and Colorectal Surgery Countess of Chester Hospital NHS Foundation Trust Chester UK

**Keywords:** colonic resection, nodal mapping, personalised treatment, splenic flexure cancers

## Abstract

**Aim:**

The optimum surgical approach to splenic flexure cancers (SFCs) remains uncertain. The aim of this survey was to explore the opinions of an international surgical community on the management and outcomes of SFC.

**Method:**

A questionnaire was constructed comprising five sections (information about respondents; definition and prognosis of SFC; operative approach; approach in specific scenarios; outcomes) and circulated through an international dissemination committee and social media.

**Results:**

The survey received 576 responses over 4 weeks across 50 countries. There was no consensus regarding the definition of the splenic flexure, whilst the proportion of respondents who did and did not think that patients with SFC had a worse outcome was equal. The overall preferred operative approach was left hemicolectomy [203 (35.2%)], followed by segmental resection [167 (29%)], extended right hemicolectomy [126 (21.9%)] and subtotal colectomy [7 (12%)]. The stated pedicles for ligation varied between resection types and also within the same resection. One hundred and sixty‐six (28.8%) respondents thought a segmental resection was associated with the worst survival and 190 (33%) thought it was associated with the best quality of life.

**Conclusion:**

This survey confirms a lack of consensus across all aspects SFC treatment. The differing approaches described are likely to represent different beliefs around the variable anatomy of this region and the associated lymphatic drainage. Future studies are required to address such inconsistencies and identify the optimum surgical strategy, whilst also incorporating quality‐of‐life metrics and patient‐reported outcomes. A one‐size‐fits‐all approach is probably not appropriate with SFC, and a more bespoke approach is required.

**What does this paper add to the literature?**

This paper demonstrates the ongoing severe lack of evidence into the management of splenic flexure cancers. It highlights gross variability, not only in the management of such patients but also in our description of colonic resections. This will need to be addressed before any meaningful comparisons can be made.

## INTRODUCTION

Carcinoma of the splenic flexure is relatively rare, accounting for only 2%–8% of colorectal cancers. The optimal surgical approach for splenic flexure cancer (SFC) is not currently defined, with numerous resection types being proposed and reported, including extended right hemicolectomy (ERH), left hemicolectomy (LH), segmental colectomy (SEG) and subtotal colectomy (STC) with or without resection of adjacent viscera such as the pancreas and spleen [1, 2, 3, 4, 5, 6].

This uncertainty and variation are likely a consequence of the variable vascular supply and associated lymphatic drainage of these tumours, which sit at the watershed area of the colon between the superior mesenteric and inferior mesenteric arterial territories [1, 4, 5, 6]. Additionally, SFCs have been reported to have increased emergency presentations with obstructive symptoms and potentially a worse prognosis [2, 3, 4, 5, 6].

Given these uncertainties, we conducted an international survey amongst surgeons to explore opinions and practices surrounding the management of SFC.

## METHOD

The questionnaire was constructed on Google Forms (Figure S1) and consisted of five sections relating to information about the respondent, their opinions of the definition of the splenic flexure and the association of SFC with poor outcomes, the operative strategy including surgical access and type of surgical resection, the surgical approach in specific scenarios, and outcomes of survival and quality of life (QoL). Respondents were asked to explain their choices or provide further comments or examples to questions as free text when appropriate. The survey was piloted locally prior to widespread dissemination and modifications were made to improve clarity of language to minimize ambiguity and to maximize the number of options available, for example adding laparoscopic‐assisted surgery as an option for the surgical approach.

The survey was targeted towards consultant/attending surgeons, surgeons in training and any other surgeons who manage splenic flexure tumours, either as part of their emergency or elective practice. To reach a wide international audience, we constructed an international dissemination committee to be responsible for disseminating the survey in their respective countries by whichever methods they felt suitable. Additionally, social media (Twitter) was used to launch and publicize the survey.

Results were summarized with descriptive statistics with categorical data presented as frequency counts and associated percentages. An alluvial diagram was constructed using RawGraphs [7] to demonstrate the relationship between surgical approach and vascular pedicles divided as stated by the respondents. Free text questions were analysed by content analysis, where free text responses of the questions were coded into categories relating to content and subcategories as required, and then summarized.

Only members of the research group have access to the data, personal identifying data were handled anonymously and will not be made public in any way; data will be kept only for the duration of the research.

## RESULTS

The survey was launched on 27 September 2021 and remained open for 4 weeks, receiving 576 responses across 50 countries (Table S1, Figure S2).

### About the respondents

The majority of respondents identified themselves as a consultant, attending or equivalent level [448 (78%)], with the next most frequent category being surgeons in training [118 (20%)]. Ten (2%) respondents identified themselves as ‘other’, including surgical fellows, speciality and specialist doctors, clinical chiefs, surgical assistants, junior doctors and retired surgeons. General and colorectal surgeons [281 (49%)] comprised the majority of respondents, with 170 (29%) describing themselves as general surgeons, 90 (16%) as colorectal surgeons and 35 (6%) as ‘other’, including surgical oncologists or surgeons of the gastrointestinal tract and other specialities such as upper gastrointestinal surgery or hepatopancreaticobiliary surgery.

### About the splenic flexure and splenic flexure cancers

#### Question: How would you define the splenic flexure segment of the colon?

The most popular definition of the splenic flexure was ‘the colonic segment between the distal third of the transverse colon and the proximal third of the descending colon’, selected by 276 (48%) respondents, but there was marked variation (Table 1). Eight (1%) proposed alternative definitions including the area of the colon associated with the spleen. A few respondents preferred descriptions relative to the vasculature, specifically describing the splenic flexure as the colonic territory between either the middle colic artery and sigmoidal arteries principally supplied by the left ascending colic artery, or the colonic territory between the left branch of the middle colic artery and the left colic artery.

**TABLE 1:** Respondents answers to the question: How would you define the splenic flexure segment of the colon?

Definition	Frequency (%)
The colonic segment extending from the distal third of the transverse colon to the first portion of the descending colon, 10 cm from the flexure	124 (21.5)
The colonic segment between the distal third of the transverse colon and the proximal third of the descending colon	276 (46.9)
The colonic segment at the splenic flexure or 10 cm proximal towards the transverse colon or 10 cm distal towards the descending colon	148 (25.7)
Uncertain	20 (3.5)
Other	8 (1.4)

#### Question: From your experience, do you think those patients presenting with colonic adenocarcinoma of the splenic flexure have worse oncological and/or survival outcomes when compared to patients presenting with adenocarcinoma elsewhere in the colon (excluding rectal tumours)?

Two hundred and forty‐one (42%) respondents answered ‘yes’, that patients presenting with colonic adenocarcinoma of the splenic flexure have worse oncological and/or survival outcomes when compared with patients presenting with adenocarcinoma elsewhere in the colon, whilst 247 (43%) answered ‘no’ and 88 (15%) were uncertain.

Of those who answered ‘yes’, 124 (52%) stated they thought it was because patients with SFCs have a greater tendency to present as an emergency, 47 (20%) stated it was because patients with SFCs have a greater tender to be ‘high risk’ (that is frail, elderly and/or comorbid patients), 103 (42.7%) stated that patients with SFCs have a greater tendency to have a more limited colonic resection performed and 185 (76.8%) felt that in patients with SFCs, there exists uncertainty regarding nodal drainage patterns, leaving the optimal resection uncertain. Four (1.7%) respondents were uncertain, whilst 14 (5.8%) stated ‘other’ reasons, including that the outcomes depend on difficulty of procedure or on the individual cancer, that right‐sided cancers do worse and that there is a higher rate of complications such as anastomotic leak in this cohort.

### Operative approach

#### Question: Which operative approach are you most likely to use for patients with an adenocarcinoma of the colon at the splenic flexure?

The majority of respondents, 338 (59%), stated they would use a laparoscopic approach, whilst 152 (26.4%) stated an open approach and 67 (12%) stated a hybrid or laparoscopic‐assisted approach would be used. Six (1%) were uncertain of their approach, whilst 13 (2.3%) stated other approaches, mostly referring to a robotic approach. A few respondents also stated that the approach would depend upon the tumour stage and/or size. Other comments included that the laparoscopic or robotic approach was considered standard, and some stated they would use the laparoscopic approach electively and an open approach in an emergency. A few respondents stated they only had open options available to them.

#### Question: What operation are you most likely to perform for patients with an adenocarcinoma of the colon at the splenic flexure?

Two hundred and three (35.2%) respondents stated they were most likely to perform a LH, whilst 167 (29%) and 126 (21.9%) stated they were most likely to perform a SEG and ERH, respectively. Only 38 (6.6%) stated they would perform a STC, 7 (1.2%) were uncertain and 35 (6.1%) stated other procedures, including an extended left hemicolectomy (ELH). Many suggested that their practice may vary depending on the anatomy, vascularization and position of the tumour. Patient factors such as age and comorbidities were also stated to influence the procedure.

#### Question: Which vasculature would you ligate during this operation for adenocarcinoma of the colon at the splenic flexure in the elective setting?

**FIGURE 1:**
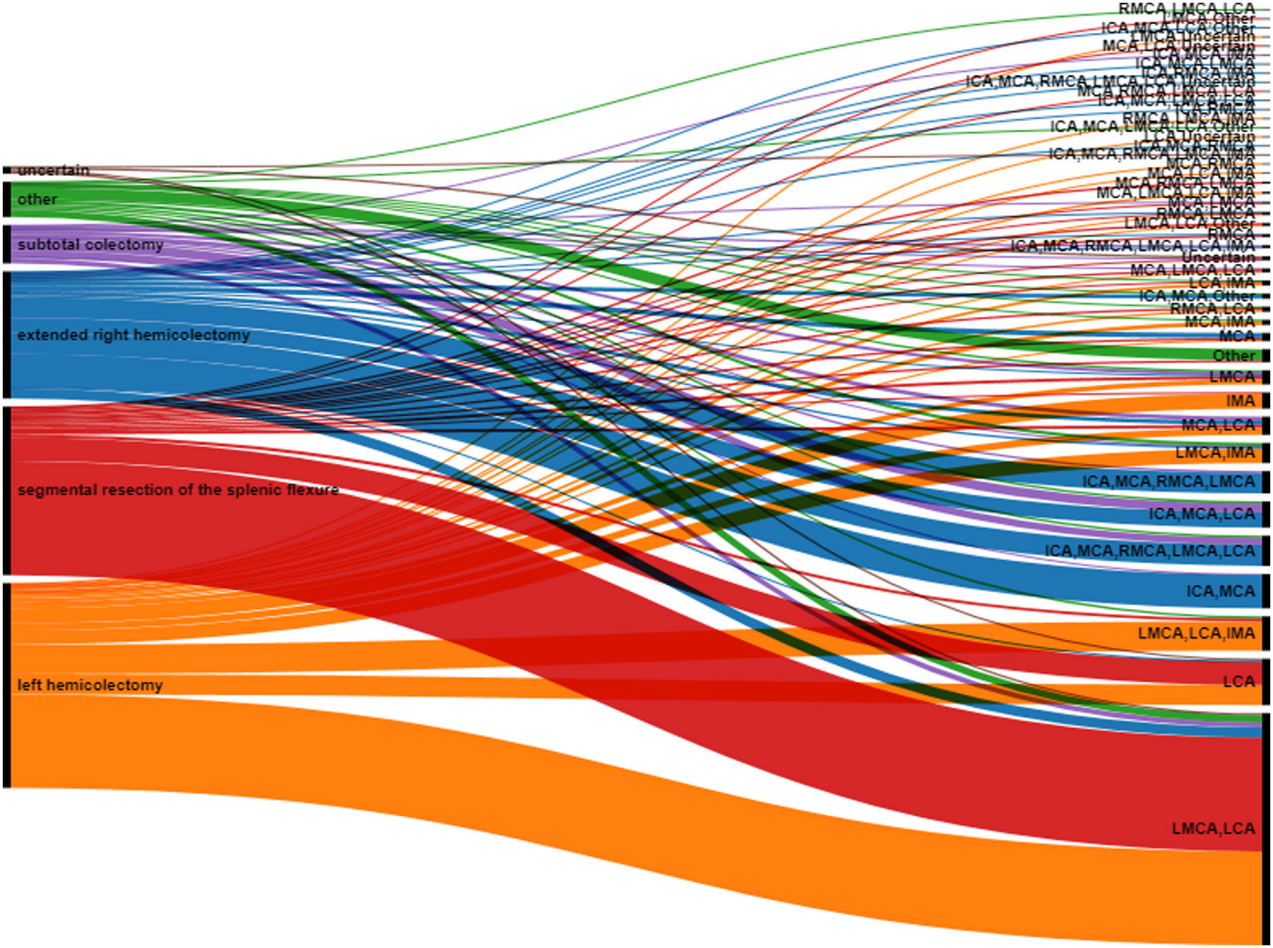
An alluvial diagram demonstrating proportional correlations between colonic resections respondents stated as their preferred approach to splenic flexure cancer (on the left) and the pedicles they describe dividing as part of that resection (on the right): ICA, ileocolic artery; IMA, inferior mesenteric artery; LCA, left colic artery; LMCA, left branch of the middle colic artery; MCA, middle colic artery; RMCA, right branch of the middle colic artery.

A variety of responses were submitted here, which not only varied between the resections the respondents had stated they were likely to perform for adenocarcinoma of the splenic flexure but also within the stated resections (Tables 2 and S2, Figure 1). Comments reinforced the need to tailor an approach based on tumour location and patient status.

**TABLE 2:** The stated resection types performed and the pedicles divided within them.

Pedicle divided	Resection, *n* (%)					
	ERH (126, 21.9%)	LH (203, 35.2%)	SEG (167, 29.0%)	STC (38, 6.6%)	Other (35, (6.1%)	Uncertain (7, 1.2%)
ICA (130, 22.6%)	101 (80.2)	0	0	22 (57.9)	6 (17.1)	1 (14.3)
MCA (173, 30.0%)	108 (85.7)	17 (8.4)	12 (7.2)	28 (73.7)	7 (20.0)	1 (14.3)
Right branch of MCA (77, 13.4%)	48 (38.1)	5 (2.5)	8 (4.8)	11 (29.0)	4 (11.4)	1 (14.3)
Left branch of MCA (379, 65.8%)	59 (46.8)	148 (72.9)	129 (77.3)	22 (57.9)	18 (51.4)	3 (42.9)
LCA (415, 72.1%)	55 (43.7)	160 (78.8)	151 (90.4)	28 (73.7)	17 (48.6)	4 (57.1)
IMA (90, 15.6%)	4 (3.2)	69 (34.0)	5 (3.0)	4 (10.5)	7 (20.0)	1 (14.3)
Other (24, 4.2%)	5 (4.0)	1 (0.5)	5 (3.0)	0	13 (37.1)	0
Uncertain (8, 1.4%)	1 (0.8)	2 (1.0)	2 (1.2)	1 (2.6)	0	2 (28.6)

Abbreviations: ERH, extended right hemicolectomy; ICA, ileocolic artery; IMA, inferior mesenteric artery; LCA, left colic artery; LH, left hemicolectomy; MCA, middle colic artery; SEG, segmental colectomy; STC, subtotal colectomy.

### Operative strategy in specific scenarios

#### Question: Might you modify your resection for a patient with adenocarcinoma of the colon at the splenic flexure who was considered to be ‘high risk’, that is frail, elderly and/or comorbid?

Three hundred and eighteen (55.2%) respondents stated ‘yes’, they would modify their resection in such a setting, 222 (38.5%) stated ‘no’, they would not modify their approach and 36 (6.3%) were uncertain if they would modify their approach in a patient who was considered to be high risk (Tables S3 and S4, Figure S3). Of those 55.2% who stated that they would modify their resection, 204 (64%) stated they would perform a SEG. The reasons offered for modifying their approach included to reduce the operating time and the risk of postoperative complications, including anastomotic leak.

#### Question: Might you modify your approach for an obstructing cancer (with viable bowel) presenting as an emergency?

The majority of respondents [375 (65%)] stated ‘yes’, they would modify their approach in the presence of an obstructing cancer, whilst 175 (31%) stated ‘no’ they would not and 25 (4%) were uncertain (Tables S5 and S6, Figure S4). Of those who said yes, they would modify their approach, 86 (23%) would have performed an ERH, 71 (19%) a STC, 53 (14%) a colonic stent, 35 (9%) a SEG, 29 (8%) a LH and 9 (2%) were uncertain of what procedure they would perform. Ninety‐three (25%) stated ‘other’, mostly expressing that they would form a stoma.

#### Question: If you answered yes, you would modify your approach for an obstructing cancer (with viable bowel) presenting as an emergency, would you most likely create a stoma?

The majority 464 (70%) stated yes, they would most likely create a stoma for an obstructing cancer.

Further details on modifications in the ‘high risk’ and an obstructing cancer can be found in Appendix S1.

### Outcomes

#### Question: Do you think the different types of colonic resections used result in different survival advantages in patients with colonic tumours of the splenic flexure?

**FIGURE 2:**
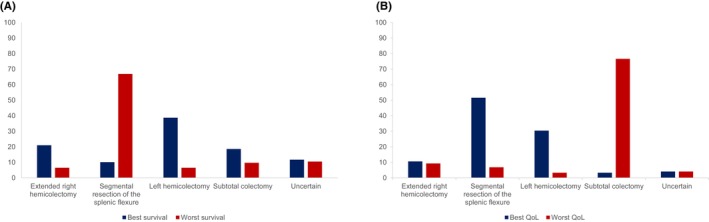
(A) Bar chart demonstrating which resection type respondents thought were associated with the best and worst survival. (B) Bar chart demonstrating which resection type respondents thought were associated with the best and worst quality of life (QoL).

Two hundred and forty‐eight (43%) respondents stated ‘yes’, they felt different types of colonic resections used in colonic tumours of the splenic flexure resulted in different survival advantages, whilst 180 (31%) did not feel they did and 148 (26%) were uncertain. The majority, 166 (67%), felt a SEG was associated with the worst survival (Figure 2). Additional comments stated that the optimum procedure remained unclear, whilst some felt that there was no difference between the procedures and a few stated that the outcome was dependent on individual circumstances.

#### Question: Do you think the different types of colonic resections used result in different quality of life?

Three hundred and sixty‐seven (64%) respondents stated ‘yes’, that different types of colonic resections were associated with different QoL, whilst 155 (27%) stated ‘no’ and 54 (9%) were uncertain. The majority of respondents, 281 (77%), felt a STC was associated with the worst QoL and 190 (52%) felt a SEG was associated with the best QoL (Figure 2). A few respondents stated there was a lack of evidence to comment, whilst some felt that there was no difference amongst the resection types.

## DISCUSSION

### Summary of findings

The response we received to this survey demonstrates excellent international interest and that the topic remains controversial, requiring further study.

Our findings demonstrate a lack of consensus on the definition of the splenic flexure segment of the colon, with respondents being equally divided as to whether tumours of the splenic flexure are thought to be associated with a worse outcome.

Although most respondents (59%) stated they would use a laparoscopic approach for SFC, there was marked heterogeneity present in the operation respondents stated they would perform. Variation in practice was also demonstrated with the wide‐ranging responses given for the vascular pedicles respondents stated they would likely divide, with variation present not only between resection types but also within resection types, demonstrating a lack of unity as to what each resection type consists of (although we acknowledge this is likely to be the case for general colonic resections, not only specific to resection of SFC).

There was a divide as to whether respondents felt different resection types resulted in different survival advantages in SFC, whilst the majority of respondents felt that different resection types were associated with differing QoL. Of those who answered ‘yes’ to these questions, LH was thought to be associated with the best survival whilst SEG was associated with the worst survival. SEG, however, was thought to be associated with the best QoL, whilst the respondents thought a STC was association with the worst QoL.

### Context of the literature

There exists a lack of clarity in guidance regarding the operative approach to splenic flexure tumours in current published recommendations [8, 9], reflecting the uncertainty in the management of these patients demonstrated in this international survey. Previous surveys have been conducted on this topic, including a 2013 UK survey and a 2017 French survey [10, 11]. In contrast to the current results showing LH to be the most popular resection type for SFC, the UK survey reported ERH as preferred (63%) and the French survey demonstrated SEG to be the most popular (70%). Both the UK and French surveys confirm our findings of great variation in practice regarding division of pedicles, even when discussing the same resection.

All three surveys had similar proportions of respondents considering SFCs to carry a worse prognosis compared with tumours at other sites, and whilst in the 2013 UK survey the majority thought it was due to delayed presentation (79%) the French survey agreed with the current results, with the majority (77%) considering it to be due to uncertainty regarding nodal draining patterns leaving the optimal resection uncertain. This uncertainty arises from the variable arterial supply of the splenic flexure, with studies reporting a number of vascular variations [12, 13, 14]. This variability in turn leads to marked variation of lymphatic drainage of this region, which needs to be considered in operative planning for SFC [15].

Few studies have attempted to address this uncertainty of nodal drainage in SFC. Watanabe et al. in a 2016 report evaluated the pattern of lymph flow in 31 patients with SFC by injecting indocyanine green adjacent to the tumour prior to laparoscopic resection and demonstrated drainage largely into the left vascular territory (left colic, left middle colic and left accessory aberrant colic arteries) [16]. Vasey et al. in 2018 utilized scintigraphic mapping with technetium‐99m in 30 patients with colonic cancers and demonstrated strong dominance of lymphatic flow (96% of patients) from the splenic flexure towards the left colic arterial territory [17]. Both these studies, however, neglected the right‐sided pedicles in their observations. Manceau et al. in 2018 reported on 65 consecutive patients undergoing a STC for SFC and found that of 18 (27%) patients with metastatic lymph nodes, 6 (33%) had involved nodes along the right colic pedicle [15]. Further larger studies, inclusive of right colonic pedicles, are clearly required to identify whether lymphatic mapping techniques are useful in tailoring the treatment of SFC.

There have been three published systematic reviews and meta‐analyses examining the approach to and outcomes of SFCs. Martínez‐Pérez in 2017 found only three comparative studies comparing ERH and LC (including both LHC and SFC) [18], Hajibandeh et al. in 2020 identified seven comparative studies and used a pair‐wise approach comparing ERH, LH and SEG [19] and in 2021 Wang et al. performed a network meta‐analysis of ten studies [20]. None of these meta‐analyses were able to demonstrate any difference in overall survival between the various resection types examined.

Since these meta‐analyses, there further observational studies have been published. A European study in 2021 examined 90 consecutive patients with SFC undergoing an emergency resection having had either an ERH, LH or SEG [21]. In 2022 a Chinese paper reported on 117 consecutive patients undergoing elective SEG, LH or ELH for SFC [22] and the French GRECCAR group utilized propensity score analysis to report outcomes between SEG, LH and STC [23]. Additionally, two studies from Italy and the USA examined outcomes between those patients undergoing a SEG compared with those undergoing extended or anatomical resection [24, 25]. None of these studies reported any difference in 5‐year overall survival between the operative groups studied.

All the studies included within the meta‐analyses were nonrandomized retrospective cohorts, largely single institution, and this is also true of subsequent published reports. Given these limitations, as well as the possibility that SFCs are heterogeneous in themselves, as demonstrated by the variable lymphatic drainage present, it is likely the studies were too biased and/or underpowered to demonstrate such findings.

The uncertainties demonstrated above are highlighted by a Delphi consensus study conducted between 2020 and 2021. This study aimed to establish a consensus on the management of SFC and after three rounds only found moderate consensus on the definition of splenic flexures and the operative technique and acknowledged that decisions were not evidence based and further work was required [26].

### Strengths and limitations

Although this survey received a very good response it is impossible to capture every single surgeon's response. There were disproportionate responses from different countries contributing to this response bias. The majority of responses were from Italy and Turkey, countries which likely had a very active dissemination committee. Some members of the dissemination committee also translated the form into their respective languages (French and Portuguese for Brazil) to maximize their responses; however, this is likely to have contributed to disproportioned responses from the different countries. Additionally, whilst we also launched the survey on Twitter to maximize responses we did not launch on any other social media site, which again may contribute to bias.

We acknowledge that the opinions and preferred stated approaches of the respondents do not necessarily indicate true practice or the quality of surgery performed. We also acknowledge that there may be further ambiguity present, as although we attempted to gauge which vascular pedicles respondents are likely to divide operatively, we did not specify the level of division and, as such, responses may not represent high ligation of the pedicle. Additionally, although a strength of the survey is that we attempted to gauge different approaches employed in varying patient scenarios for SFC, such decision‐making in real life is multifactorial and we accept that it is impossible to truly capture such situations by way of a survey.

### Clinical implications and future work

It is clear the optimal surgical approach to SFC remains elusive, with numerous proposed strategies and rationales and the proposed resections themselves seemingly not standardized. Whether SFCs are associated with worse outcomes when compared with other colonic tumours also needs to be clarified and, if so, whether it is the inherent tumour biology, the presentation of SFC or, as suggested by the results of this survey, our lack of understanding of its anatomy and as such our inability to provide the appropriate treatment. As a result, we are currently unable to deliver standardized and optimum care to this cohort.

These concepts need to be standardized to facilitate comparisons. Future prospective studies will be required to investigate the uncertainty and variability of the arterial blood supply and lymphatic drainage pattern of SFCs and subsequently the optimum surgical strategy. It may be that a one‐size‐fits‐all approach is not appropriate and more careful consideration of individual anatomy for these tumours and a personalized surgical approach may be required.

The splenic flexure is an operatively challenging area of anatomy, with its proximity to the spleen and short mesentery, demonstrated by the exclusion of transverse and splenic flexure cancers from the national colonic laparoscopic trials [27, 28, 29]. It is well established in rectal cancer that the quality of the pathological specimen relates to outcome and there is evidence for this relationship in colonic procedures [22, 30, 31]. Future studies should also examine the quality of the surgical specimen as a potential contributor to outcomes and an area that could be improved.

The results of this survey demonstrated the opinion that there is a trade‐off to be had between the extent of surgical resection to achieve a good oncological outcome and the resultant QoL. It is essential that any future study examining the surgical approach to SFC not only examines oncological outcomes but also postresection QoL and gastrointestinal function alongside patient‐reported outcomes.

## AUTHOR CONTRIBUTIONS

**H. Sekhar:** Conceptualization; investigation; writing – original draft; methodology; writing – review and editing; formal analysis; data curation. **M. Dyer:** Writing – review and editing; writing – original draft; data curation; formal analysis. **M. Khan:** Writing – review and editing; investigation. **P.J. Mitchell:** Investigation; writing – review and editing. **N.P. West:** Conceptualization; investigation; writing – review and editing; methodology. **S. Moug:** Conceptualization; investigation; methodology; writing – review and editing. **D. Vimalachandran:** Investigation; conceptualization; methodology; writing – review and editing.

## FUNDING INFORMATION

No funding was required for this study.

## CONFLICT OF INTEREST STATEMENT

All authors have no conflicts of interest to declare.

## ETHICS STATEMENT

This study did not involve any patients or patient material and as such did not require any ethics approvals.

## Supporting information


Appendix S1

## Data Availability

The data that support the findings of this study are available from the corresponding author upon reasonable request.

## INTERNATIONAL DISSEMINATION COMMITTEE

Algeria: A. Tidjane. Argentina: Argentina Coloproctology Society. Australia and New Zealand: N. Dudi‐Venkata, H. Mohan, T. Sammour. Azerbaijan: E. Samadov. Belgium: G. Van Ramshorst. Brazil: G.M. Gomes, G.A. Laporte. Bulgaria: M. Slavchev. China: D. O'Reilly. Egypt: I. Sallam, M. Shalaby. France: E. Duchalais, B. Seeliger. Germany: W. Hohenberger, U. Ronellenfitsch. Ghana: F. Gyamfi. Greece: I. Katsoulis, K. Stamou. India: A. Mehuj, S. Rajan. Ireland: J. Larkin, E. Ryan. Italy: E. Baldini, M. Campanell, G. Canonico, G. Capolupo, M. Caricato, F. Colombo, P. DeNardi, C. Feo, R. Galleano, P. Lapolla, G. Lisi, V. Lizzi, D. Matteo, S. Novello, F. Pata, T. Perra, E. Pinotti, M. Romano, F. Rosa, M. Rottoli, P. Sileri, A. Taddei, L. Tirloni. Japan: H. Ueno. Jordan: F. Fa. Lithuania: A. Dulskas, N. Samalavicius. Morocco: A. Souadka. Mexico: M. Benitez, E. Divan, C. Soule. The Netherlands: H. Kroon. Nigeria: A. Adeyeye, J.G. Makama. Norway: H.L. Thorsen. Romania: F. Grama, I. Negoi. Russia: A. Vardanyan. South Africa: South African Colorectal Society. Spain: J. Escartin, M. Estaire‐Gómez, M.H. Garcia, F. Mendoza‐Moreno, M. Prats. Sweden: M. Rutegard. Turkey: S. Atici, E. Colak, M. Tanal. USA: D. Keller, S. Wexner.

## COLLABORATORS

Algeria: N. Djelali, A. Hebbar, S. Mesli. Australia: Y. Arafat, N. Blefari, T. Chittleborough, Y.H. Lam, S. Stevens. Belgium: E. van Eetvelde. Brazil: A. Awad, M. Coura, C. da Silva Cardial, M. Ebrahim, H. Elfeki, D. Gill, A. Sakr, F.M. Vieira. France: A. Castaldi, J. Loriau, C. Niki, M. Pocard, N. Regent, J.J. Tuech. Ghana: R. Awoonor‐Williams. Greece: M. Frountazas, N. Michalopoulos, D. Schizas, T. Sidiropoulos, M. Sotiropoulou, A. Syllaios, P. Vassiliu. Ireland: R. Cahill, M. Flanagan, M. Kelly, N. Lynch, L. O'Connell, J. Ryan. Italy: G. Alemanno, M. Ardu, G. Cerino, G. Gallo, F. Ghignone, I. Iannone, A. Mingoli, I. Montroni, T. Nelli, G. Pellino, M.V. Papa, A. Porcu, D. Sasia, S. Di Saverio, N. Tamini, C. Tanda, P. Ziprin, M. Piccoli. Lithuania: D. Venskutonis. Malaysia: M. Thanapal. Mexico: M. Trego‐Avila. Morocco: M. Anass, M. Hamid. New Zealand: A. Lin. Nigeria: A. Adejumo, O. Adewunmi, O. Adeyemi, B. Aminu, I. Chukwu, I. Garzali, S. Irmiya. Pakistan: N.A. Khan. Peru: H.B. Hernandez. Portugal: N. Figueiredo. Romania: E.A. Bonci, O. Gingina, S.T. Makkai‐Popa, A.M. Musina. South Africa: D. Lutrin, S. Sifuba. Spain: J.M. Alegre, M. Alvarez‐Gallego, P. Baños, V. Carneros, C.J. Gómez‐Díaz, S. Jeri‐McFarlane, B. Matias, C. Placer‐Galán, P. Tejedor, V. Vigorita. Sweden: K. Tsimogiannis. Turkey: C. Akyol, O. Bozbiyik, O. Duzgun, E. Kamer, A. Isik, S. Leventoglu, N. Okkabaz, I. Ozata, V. Ozben, A. Ozcan, O. Ozkan, A. Şanli, E. Sivrikoz, H. Ulgur. Tunisia: P. Okeny. UK: P. Barrow, A. Beggs, A. Bhowmick, S. Chapman, J. Davies, G. Faulkner, N. Heywood, A. Renwick, K. Sahnan, M.H. Siddique, C. Smart, P. Sutton, D. Watt, L.W. Wheldon. USA: M. Whiteford.

## References

[1] Binda GA , Amato A , Alberton G , Bruzzone M , Secondo P , Lòpez‐Borao J , et al. Surgical treatment of a colon neoplasm of the splenic flexure: a multicentric study of short‐term outcomes. Colorectal Dis. 2020;22(2):146–153. 31454443 10.1111/codi.14832

[2] de'Angelis N , Martínez‐Pérez A , Winter DC , Landi F , Vitali GC , Le Roy B , et al. Extended right colectomy, left colectomy, or segmental left colectomy for splenic flexure carcinomas: a European multicenter propensity score matching analysis. Surg Endosc. 2021;35(2):661–672. 32072288 10.1007/s00464-020-07431-9

[3] Martín Arévalo J , Moro‐Valdezate D , García‐Botello SA , Pla‐Martí V , Garcés‐Albir M , Pérez Santiago L , et al. Propensity score analysis of postoperative and oncological outcomes after surgical treatment for splenic flexure colon cancer. Int J Colorectal Dis. 2018;33(9):1201–1213. 29845387 10.1007/s00384-018-3063-1

[4] Nakagoe T , Sawai T , Tsuji T , Jibiki M , Ohbatake M , Nanashima A , et al. Surgical treatment and subsequent outcome of patients with carcinoma of the splenic flexure. Surg Today. 2001;31(3):204–209. 11318121 10.1007/s005950170169

[5] Odermatt M , Siddiqi N , Johns R , Miskovic D , Khan O , Khan J , et al. Short‐ and long‐term outcomes for patients with splenic flexure tumours treated by left versus extended right colectomy are comparable: a retrospective analysis. Surg Today. 2014;44(11):2045–2051. 24306213 10.1007/s00595-013-0803-2

[6] Rega D , Pace U , Scala D , Chiodini P , Granata V , Fares Bucci A , et al. Treatment of splenic flexure colon cancer: a comparison of three different surgical procedures: experience of a high volume cancer center. Sci Rep. 2019;9(1):10953. 31358904 10.1038/s41598-019-47548-z PMC6662908

[7] Mauri M , Elli T , Caviglia G , Uboldi G , Azzi M . Proceedings of the 12th Biannual Conference on Italian SIGCHI Chapter. RAWGraphs: A Visualisation Platform to Create Open Outputs. Cagliari, Italy: Association for Computing Machinery; 2017. p. 28.

[8] Moran B , Cunningham C , Singh T , Sagar P , Bradbury J , Geh I , et al. Association of Coloproctology of Great Britain & Ireland (ACPGBI): guidelines for the management of cancer of the colon, rectum and anus (2017) – surgical management. Colorectal Dis. 2017;19(S1):18–36. 28632309 10.1111/codi.13704

[9] Vogel JD , Felder SI , Bhama AR , Hawkins AT , Langenfeld SJ , Shaffer VO , et al. The American Society of Colon and Rectal Surgeons clinical practice guidelines for the management of colon cancer. Dis Colon Rectum. 2022;65(2):148–177. 34775402 10.1097/DCR.0000000000002323

[10] Chan D , Shah P , Saklani A . Current trends and controversies in the management of patients with splenic flexure tumours. J Cancer Res Ther. 2013;1:8–10.

[11] Manceau G , Benoist S , Panis Y , Rault A , Mathonnet M , Goere D , et al. Elective surgery for tumours of the splenic flexure: a French inter‐group (AFC, SFCD, FRENCH, GRECCAR) survey. Tech Coloproctol. 2020;24(2):191–198. 31939046 10.1007/s10151-019-02143-2

[12] Griffiths JD . Surgical anatomy of the blood supply of the distal colon. Ann R Coll Surg Engl. 1956;19(4):241–256. 13363265 PMC2378072

[13] Fukuoka A , Sasaki T , Tsukikawa S , Miyajima N , Ostubo T . Evaluating distribution of the left branch of the middle colic artery and the left colic artery by CT angiography and colonography to classify blood supply to the splenic flexure. Asian J Endosc Surg. 2017;10(2):148–153. 28008722 10.1111/ases.12349

[14] Cheruiyot I , Cirocchi R , Munguti J , Davies RJ , Randolph J , Ndung'u B , et al. Surgical anatomy of the accessory middle colic artery: a meta‐analysis with implications for splenic flexure cancer surgery. Colorectal Dis. 2021;23(7):1712–1720. 33721386 10.1111/codi.15630

[15] Manceau G , Mori A , Bardier A , Augustin J , Breton S , Vaillant JC , et al. Lymph node metastases in splenic flexure colon cancer: is subtotal colectomy warranted? J Surg Oncol. 2018;118(6):1027–1033. 30212600 10.1002/jso.25169

[16] Watanabe J , Ota M , Suwa Y , Ishibe A , Masui H , Nagahori K . Evaluation of lymph flow patterns in splenic flexural colon cancers using laparoscopic real‐time indocyanine green fluorescence imaging. Int J Colorectal Dis. 2017;32(2):201–207. 27695977 10.1007/s00384-016-2669-4

[17] Vasey CE , Rajaratnam S , O'Grady G , Hulme‐Moir M . Lymphatic drainage of the splenic flexure defined by intraoperative scintigraphic mapping. Dis Colon Rectum. 2018;61(4):441–446. 29521825 10.1097/DCR.0000000000000986

[18] Martínez‐Pérez A , Brunetti F , Vitali GC , Abdalla S , Ris F , de'Angelis N . Surgical treatment of colon cancer of the splenic flexure: a systematic review and meta‐analysis. Surg Laparosc Endosc Percutan Tech. 2017;27(5):318–327. 28796653 10.1097/SLE.0000000000000419

[19] Hajibandeh S , Hajibandeh S , Hussain I , Zubairu A , Akbar F , Maw A . Comparison of extended right hemicolectomy, left hemicolectomy and segmental colectomy for splenic flexure colon cancer: a systematic review and meta‐analysis. Colorectal Dis. 2020;22(12):1885–1907. 32757361 10.1111/codi.15292

[20] Wang X , Zheng Z , Chen M , Lu X , Huang S , Huang Y , et al. Subtotal colectomy, extended right hemicolectomy, left hemicolectomy, or splenic flexure colectomy for splenic flexure tumors: a network meta‐analysis. Int J Colorectal Dis. 2021;36(2):311–322. 32975595 10.1007/s00384-020-03763-z

[21] de'Angelis N , Espin E , Ris F , Landi F , Le Roy B , Coccolini F , et al. Emergency surgery for splenic flexure cancer: results of the SFC study group database. World J Emerg Surg. 2021;16(1):20. 33926504 10.1186/s13017-021-00365-0 PMC8086132

[22] Huang M , Wang X , Shao Y , Huang S , Huang Y , Chi P . Surgical treatment of splenic flexure colon cancer: analysis of short‐term and long‐term outcomes of three different surgical procedures. Front Oncol. 2022;12:884484. 35814379 10.3389/fonc.2022.884484 PMC9263504

[23] Manceau G , Alves A , Meillat H , Benhaïm L , Ouaïssi M , Panis YH , et al. What is the optimal elective colectomy for splenic flexure cancer: end of the debate? A multicenter study from the GRECCAR group with a propensity score analysis. Dis Colon Rectum. 2022;65(1):55–65. 34882628 10.1097/DCR.0000000000001937

[24] Degiuli M , Reddavid R , Ricceri F , di Candido F , Ortenzi M , Elmore U , et al. Segmental colonic resection is a safe and effective treatment option for colon cancer of the splenic flexure: a Nationwide retrospective study of the Italian Society of Surgical Oncology–Colorectal Cancer Network Collaborative Group. Dis Colon Rectum. 2020;63(10):1372–1382. 32969880 10.1097/DCR.0000000000001743

[25] Zhang C , Calderon E , Chang YH , Han GR , Kelley SR , Merchea A , et al. Short and long‐term oncologic outcomes of patients with colon cancer of the splenic flexure. Am J Surg. 2023;226(1):77–82. 36858866 10.1016/j.amjsurg.2023.02.005

[26] Benlice C , Parvaiz A , Baca B , Hohenberger W , Miskovic D , Stocchi L , et al. Standardization of the definition and surgical management of splenic flexure carcinoma by an international expert consensus using the Delphi technique: room for improvement? Dis Colon Rectum. 2023;66(6):805––15. 36716403 10.1097/DCR.0000000000002692

[27] Colon Cancer Laparoscopic or Open Resection Study Group , Buunen M , Veldkamp R , Hop WC , Kuhry E , Jeekel J , et al. Survival after laparoscopic surgery versus open surgery for colon cancer: long‐term outcome of a randomised clinical trial. Lancet Oncol. 2009;10(1):44–52. 19071061 10.1016/S1470-2045(08)70310-3

[28] Clinical Outcomes of Surgical Therapy Study Group , Nelson H , Sargent DJ , Wieand HS , Fleshman J , Anvari M , et al. A comparison of laparoscopically assisted and open colectomy for colon cancer. N Engl J Med. 2004;350(20):2050–2059. 15141043 10.1056/NEJMoa032651

[29] Guillou PJ , Quirke P , Thorpe H , Walker J , Jayne DG , Smith AM , et al. Short‐term endpoints of conventional versus laparoscopic‐assisted surgery in patients with colorectal cancer (MRC CLASICC trial): multicentre, randomised controlled trial. Lancet. 2005;365(9472):1718–1726. 15894098 10.1016/S0140-6736(05)66545-2

[30] West NP , Morris EJ , Rotimi O , Cairns A , Finan PJ , Quirke P . Pathology grading of colon cancer surgical resection and its association with survival: a retrospective observational study. Lancet Oncol. 2008;9(9):857–865. 18667357 10.1016/S1470-2045(08)70181-5

[31] Quirke P , Steele R , Monson J , Grieve R , Khanna S , Couture J , et al. Effect of the plane of surgery achieved on local recurrence in patients with operable rectal cancer: a prospective study using data from the MRC CR07 and NCIC‐CTG CO16 randomised clinical trial. Lancet. 2009;373(9666):821–828. 19269520 10.1016/S0140-6736(09)60485-2 PMC2668948

