# Supplementary material for: SF‐CORNER (splenic flexure colorectal cancer): an international survey of operative approaches and outcomes for cancers of the splenic flexure
Source: Colorectal Dis. 2024 Feb 12;26(4):660–8. doi: 10.1111/codi.16895 (PMC12150829; doi:10.1111/codi.16895)
Supplement: Supplementary file 1 — Appendix S1 [file CODI-26-660-s001.docx]

**Supplementary material**

**SF-CORNER (Splenic Flexure COloRectal caNcER): An international survey of practice of operative approaches and outcomes for cancers of the splenic flexure**

**Sekhar et al.**

**THE DISTRIBUTED SURVEY**


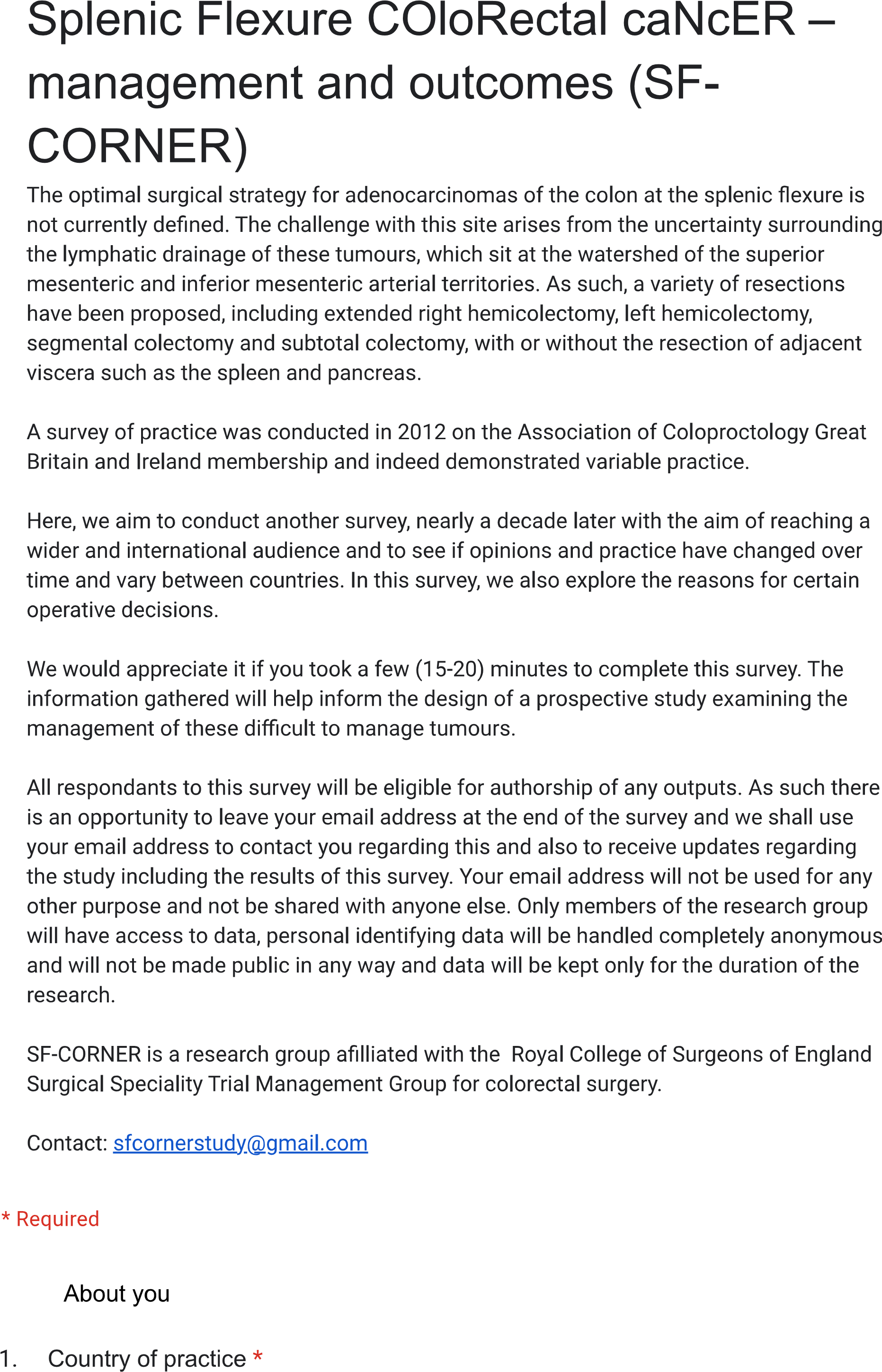
Figure S1: A copy of the distributed survey.


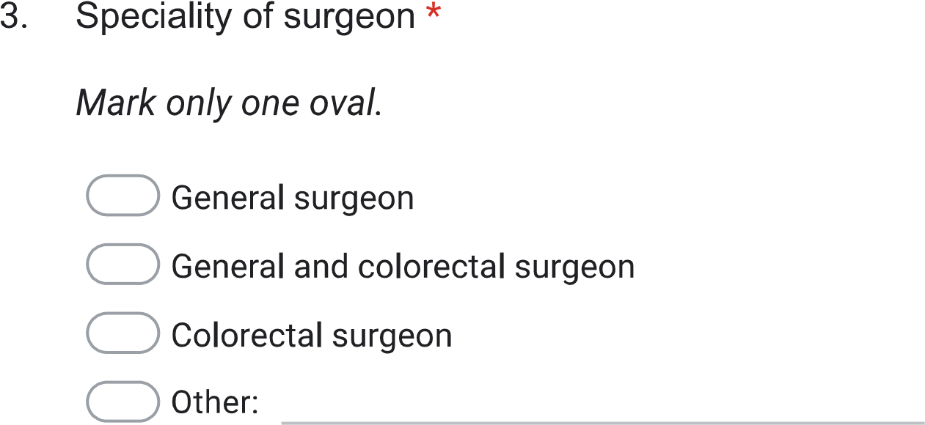


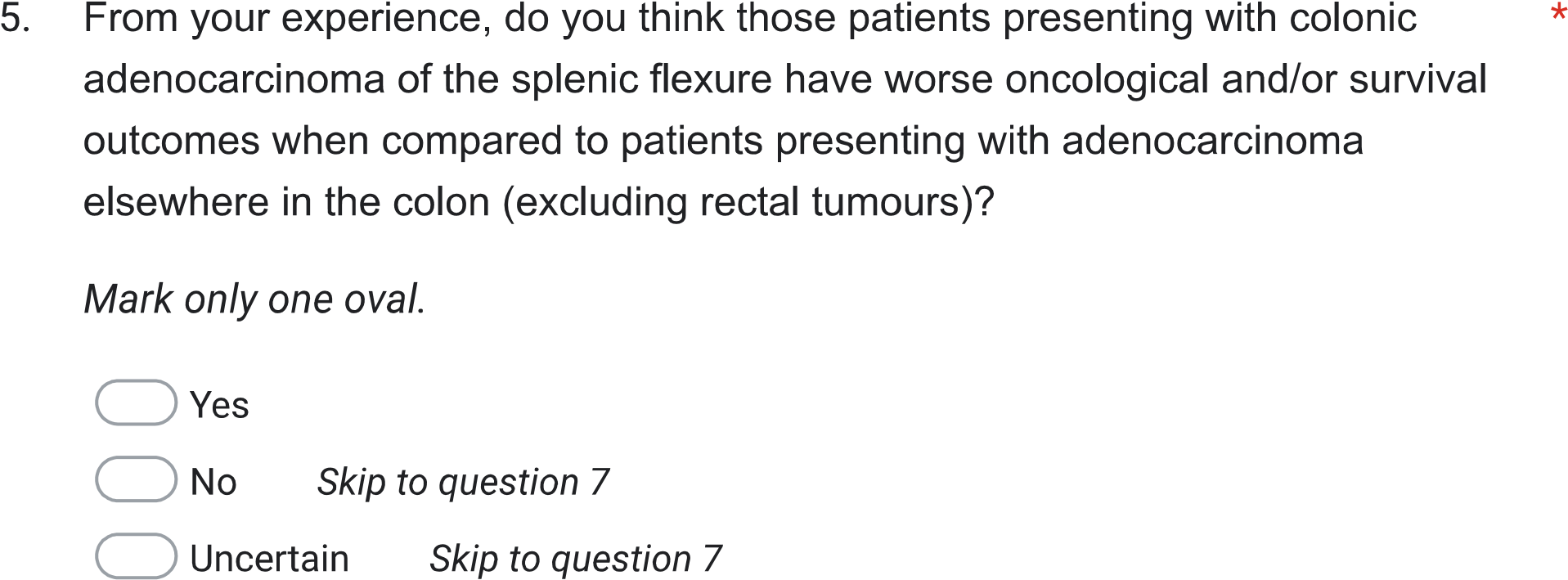

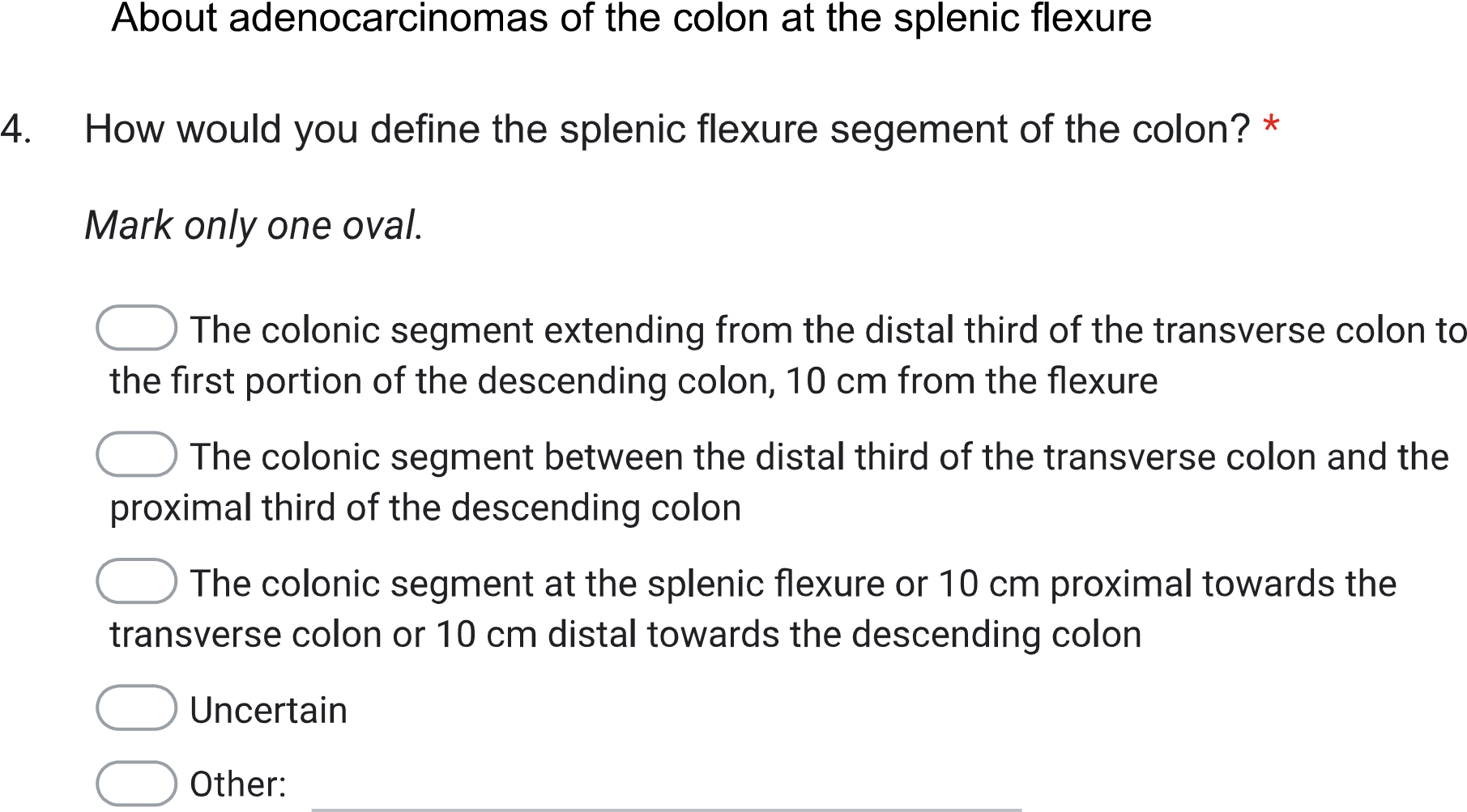


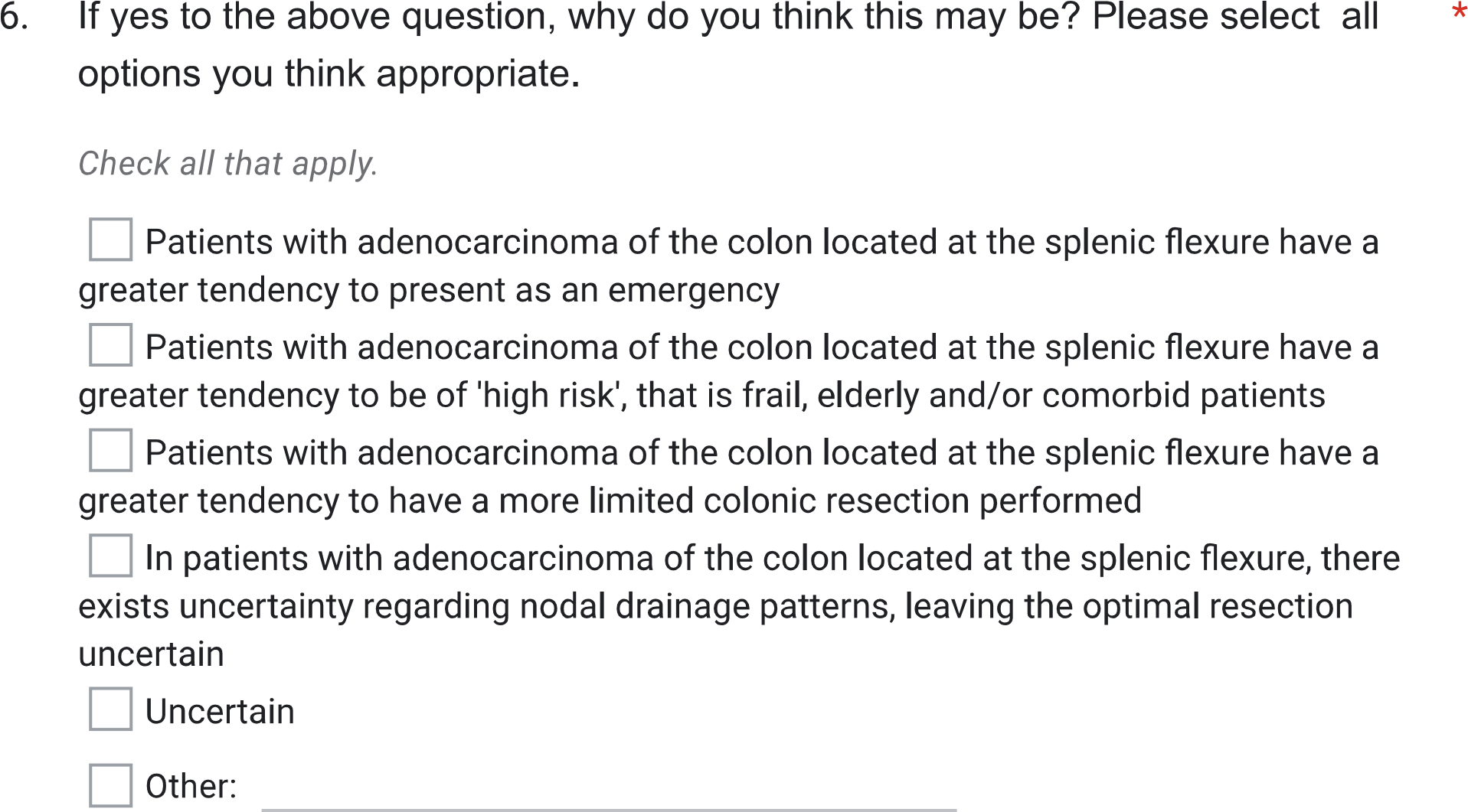


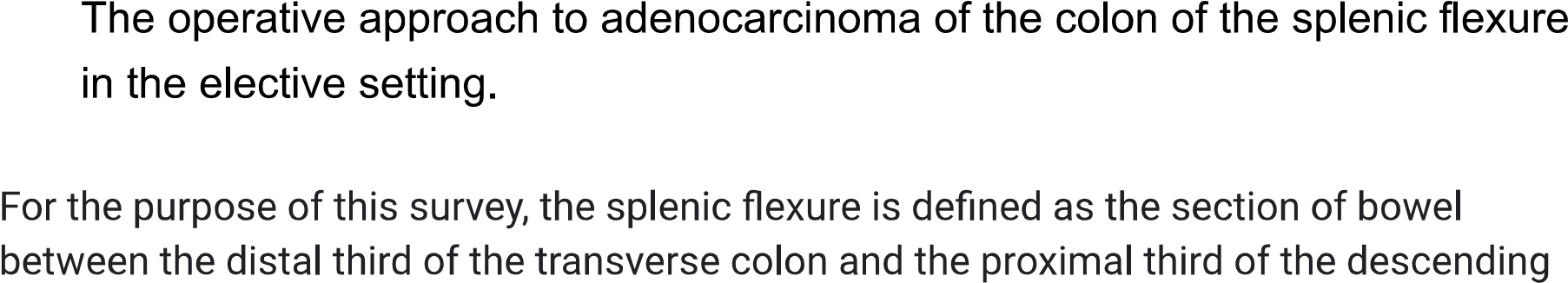


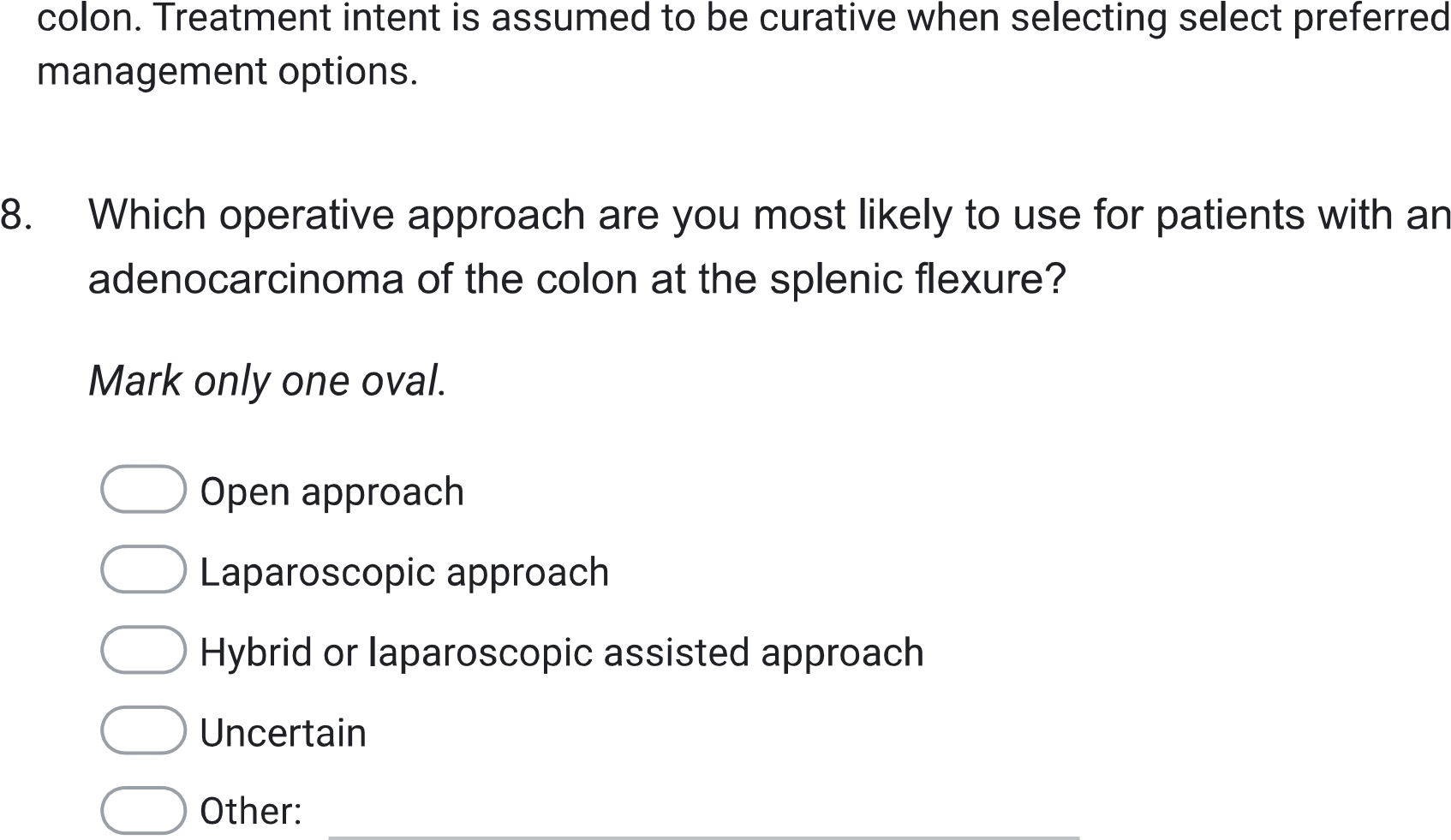


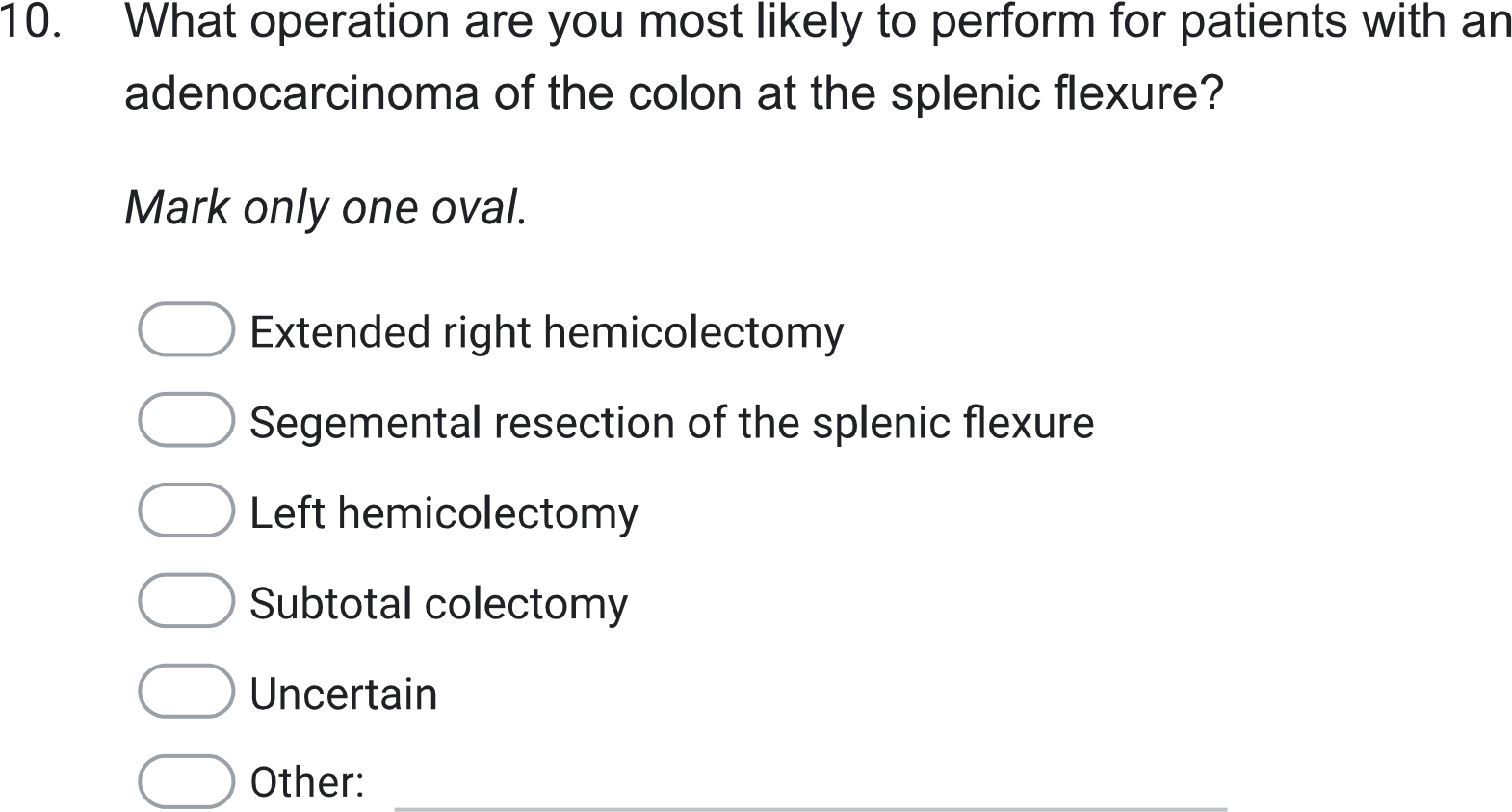


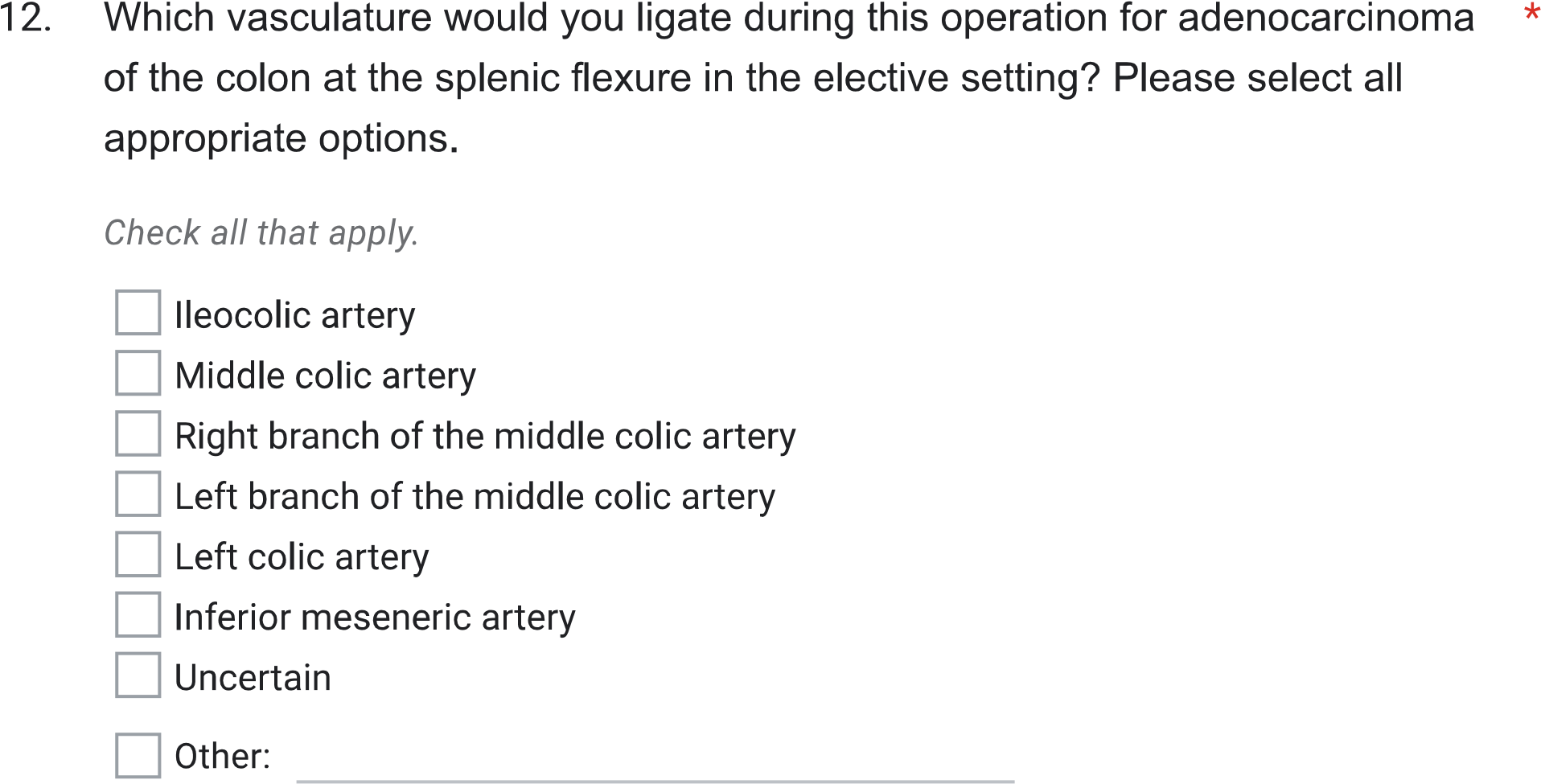


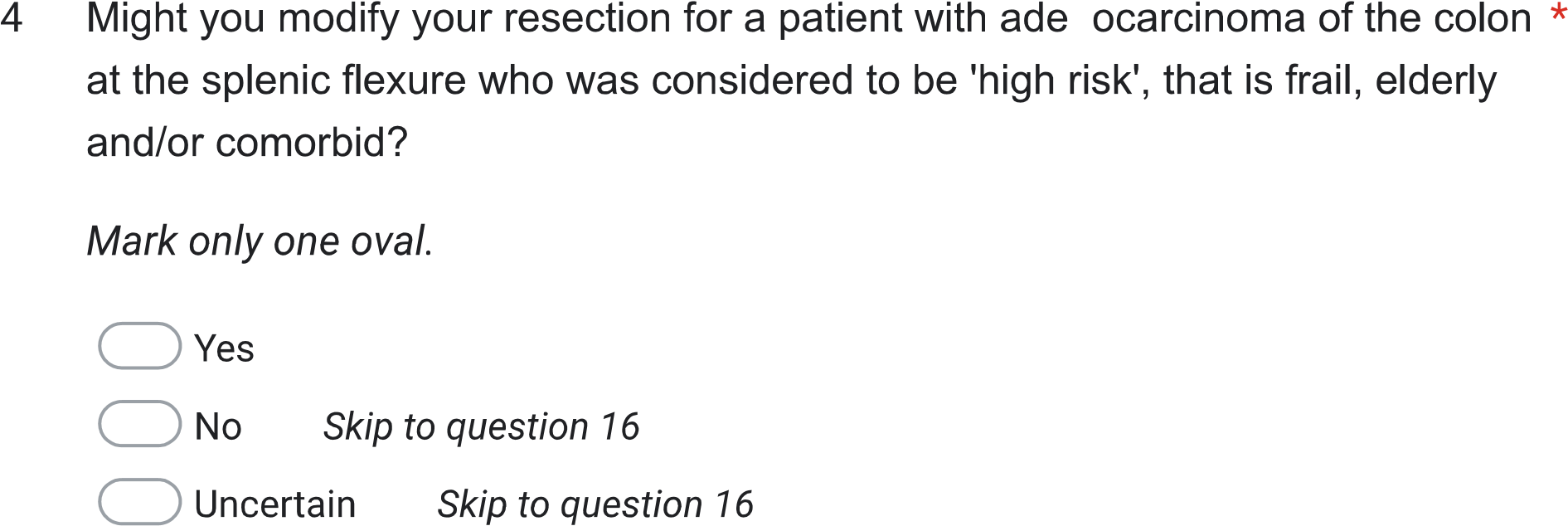


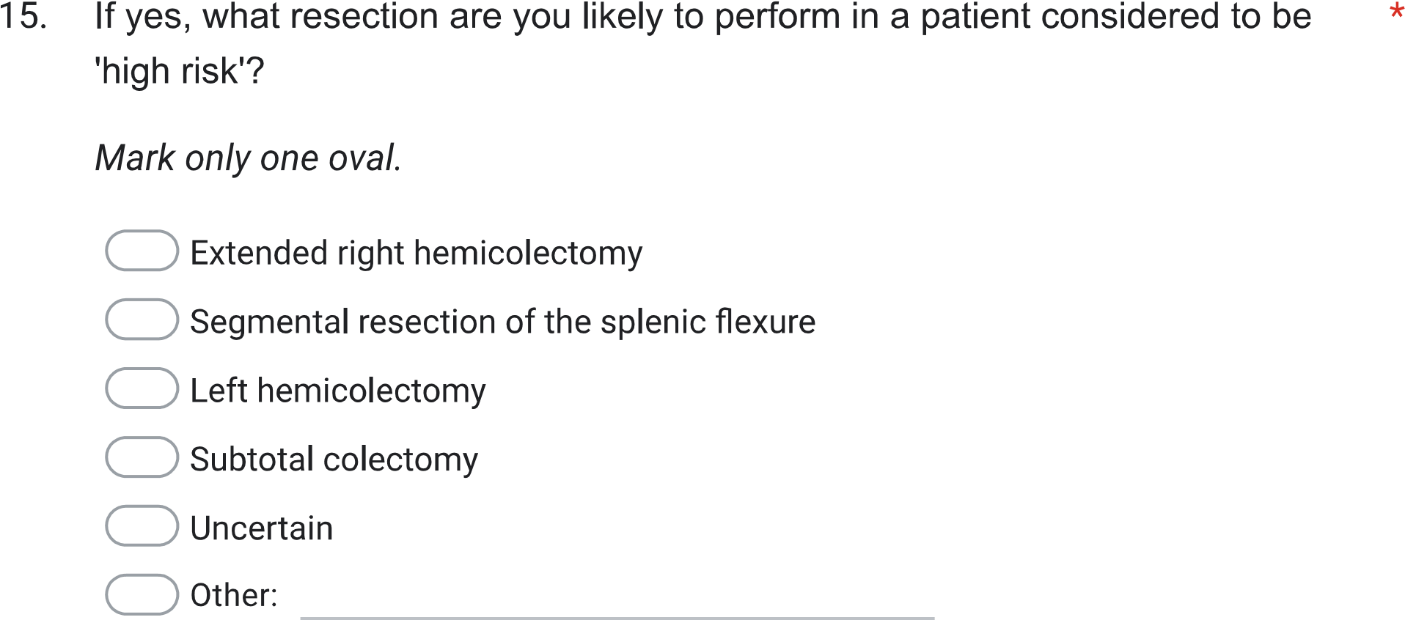


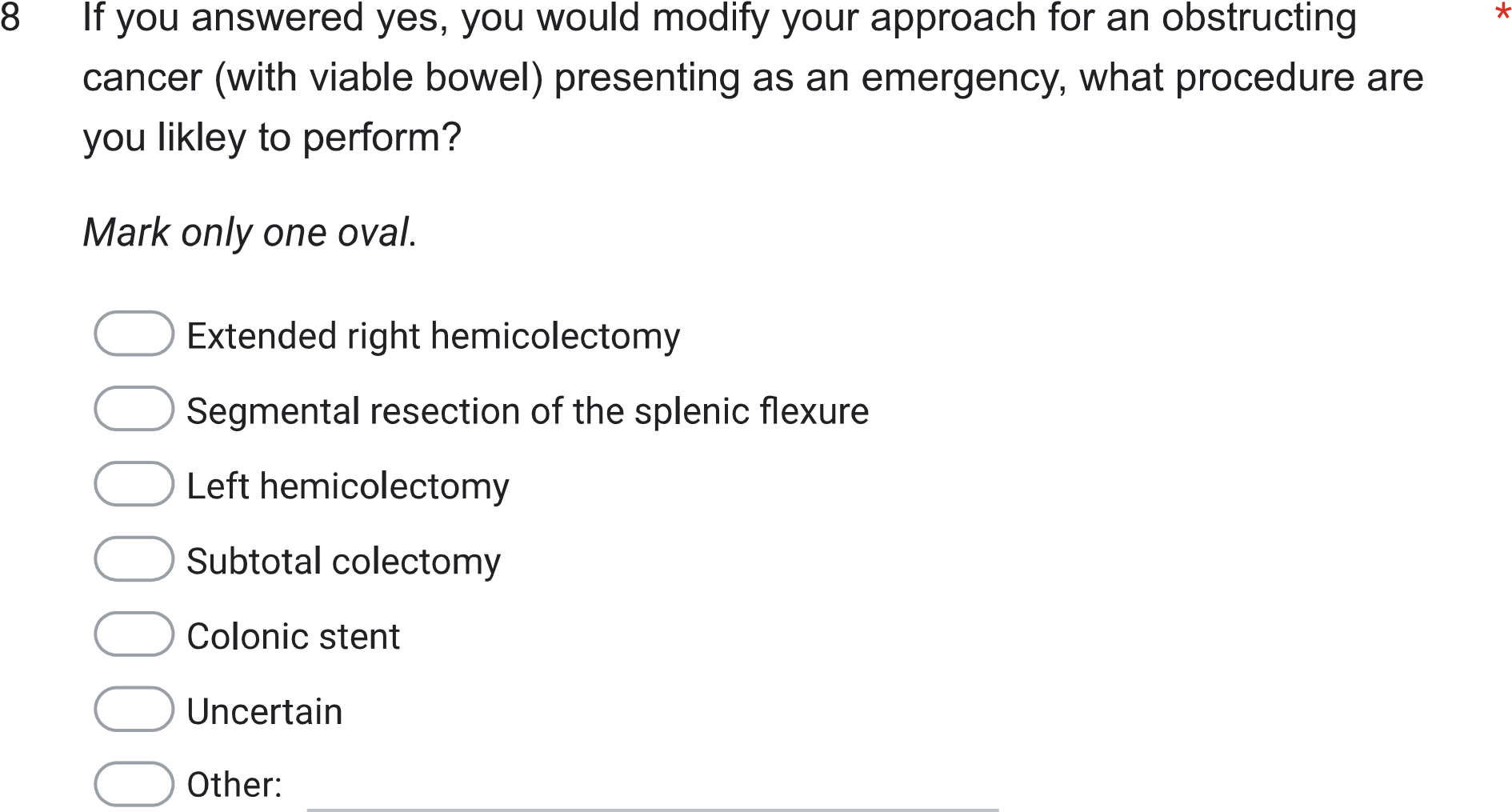

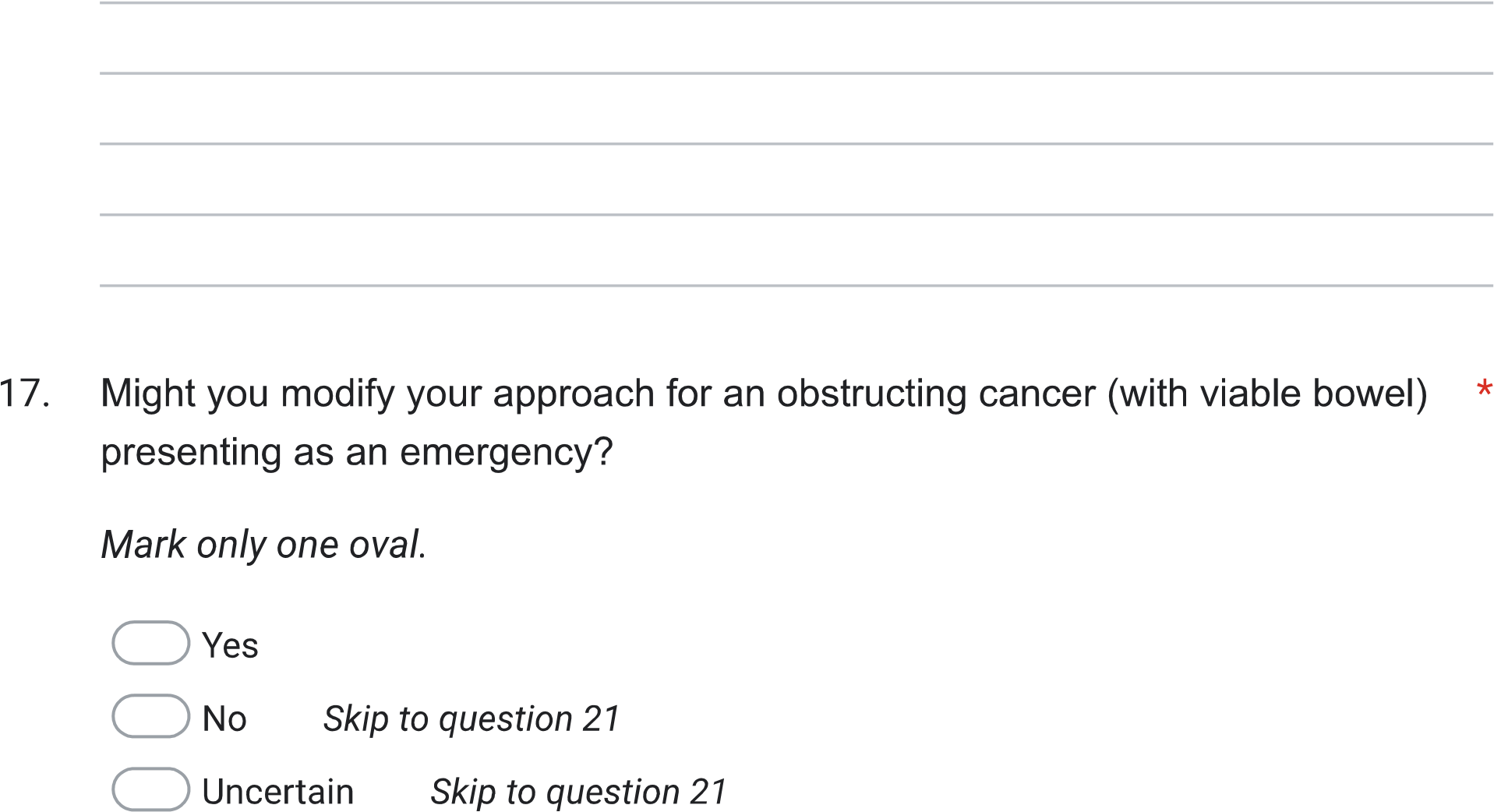


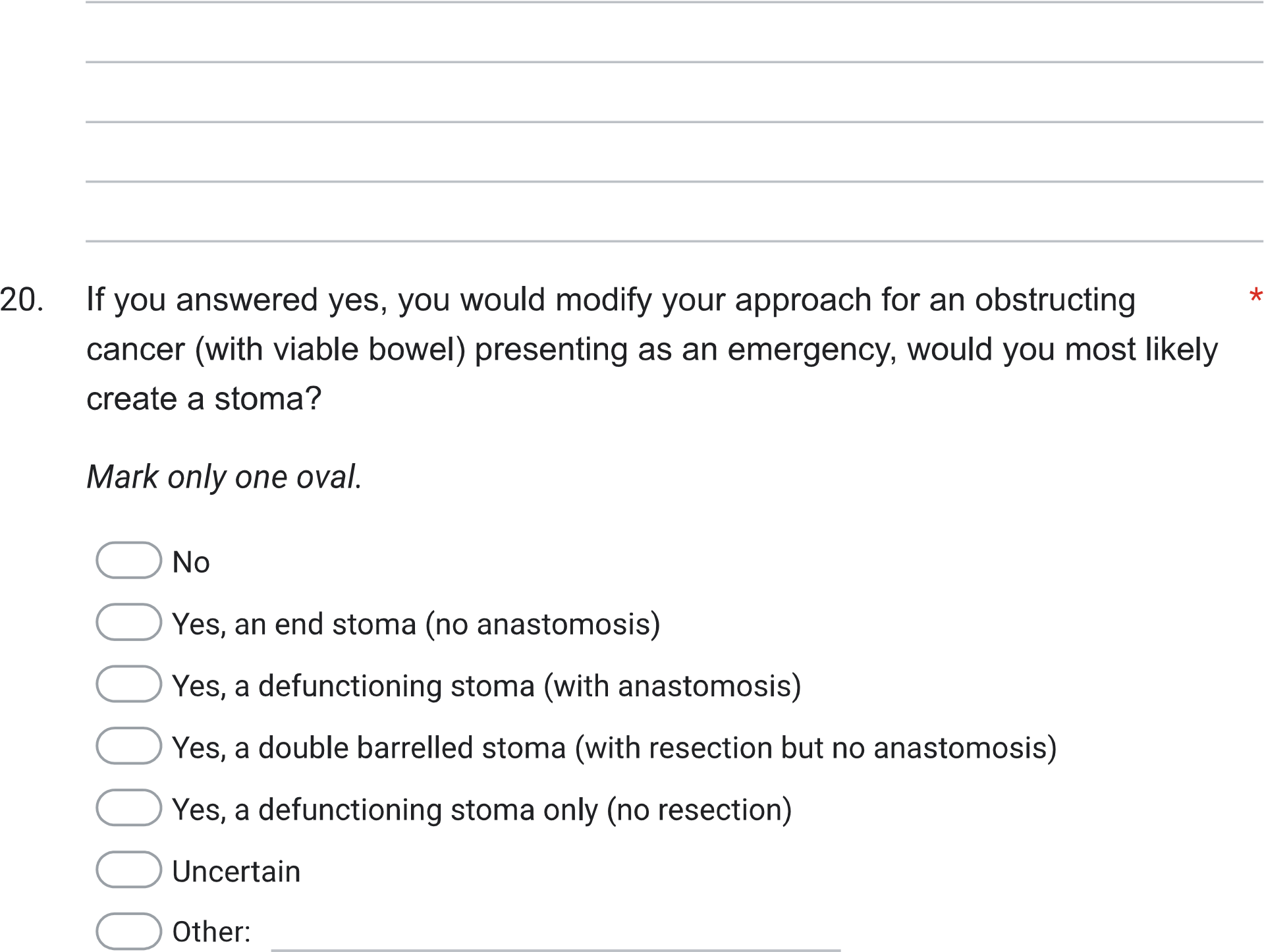


21. Please explain your answer or provide further comments or examples:


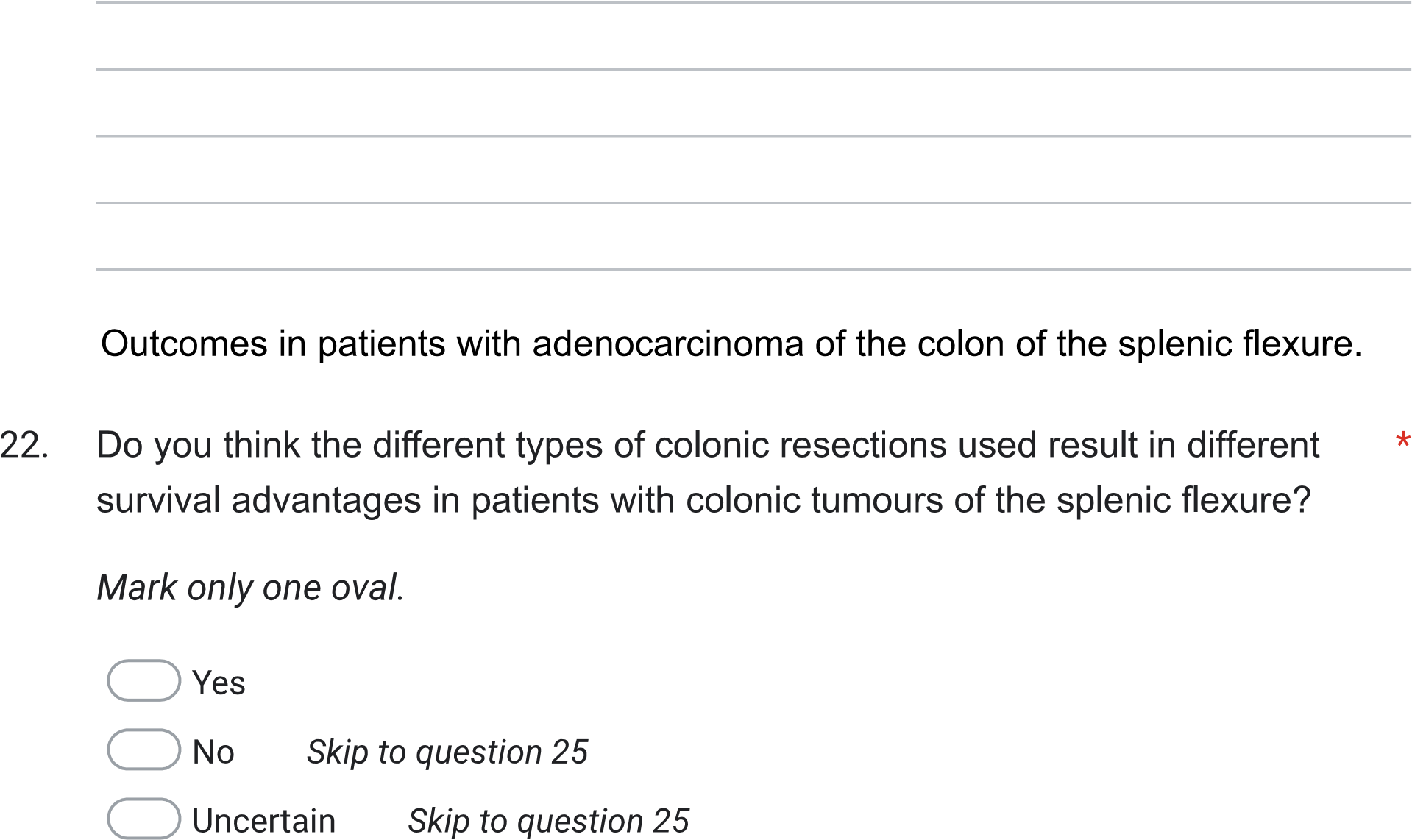

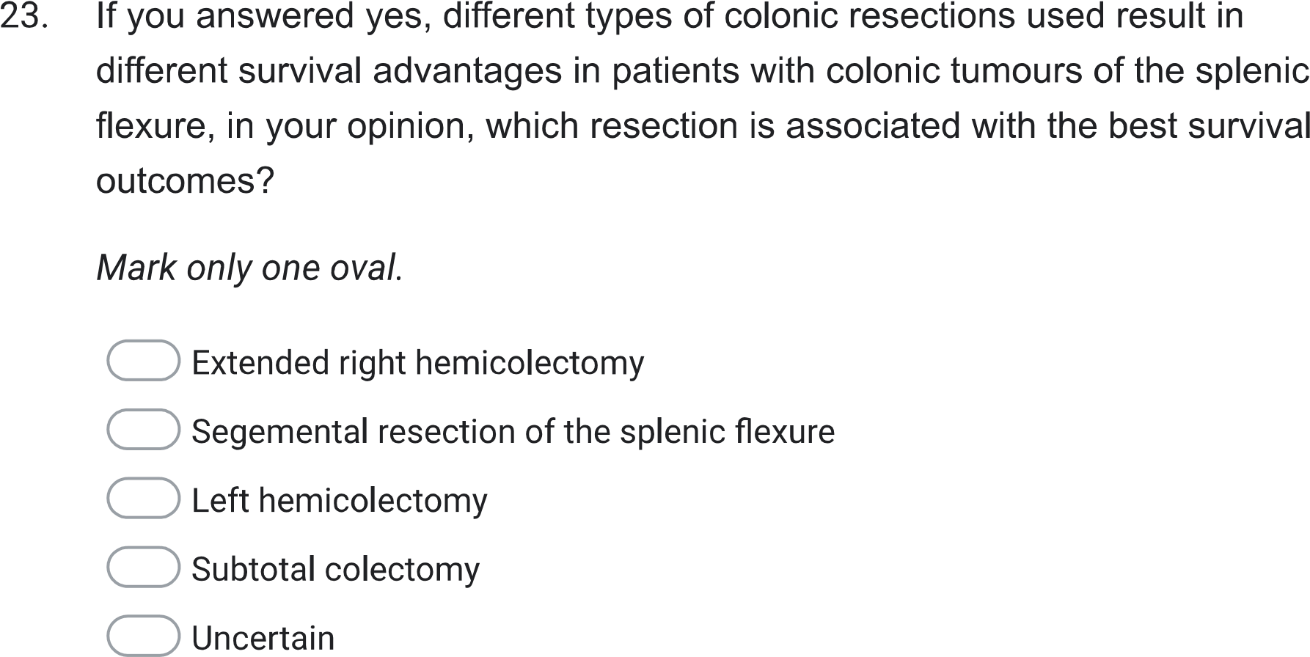


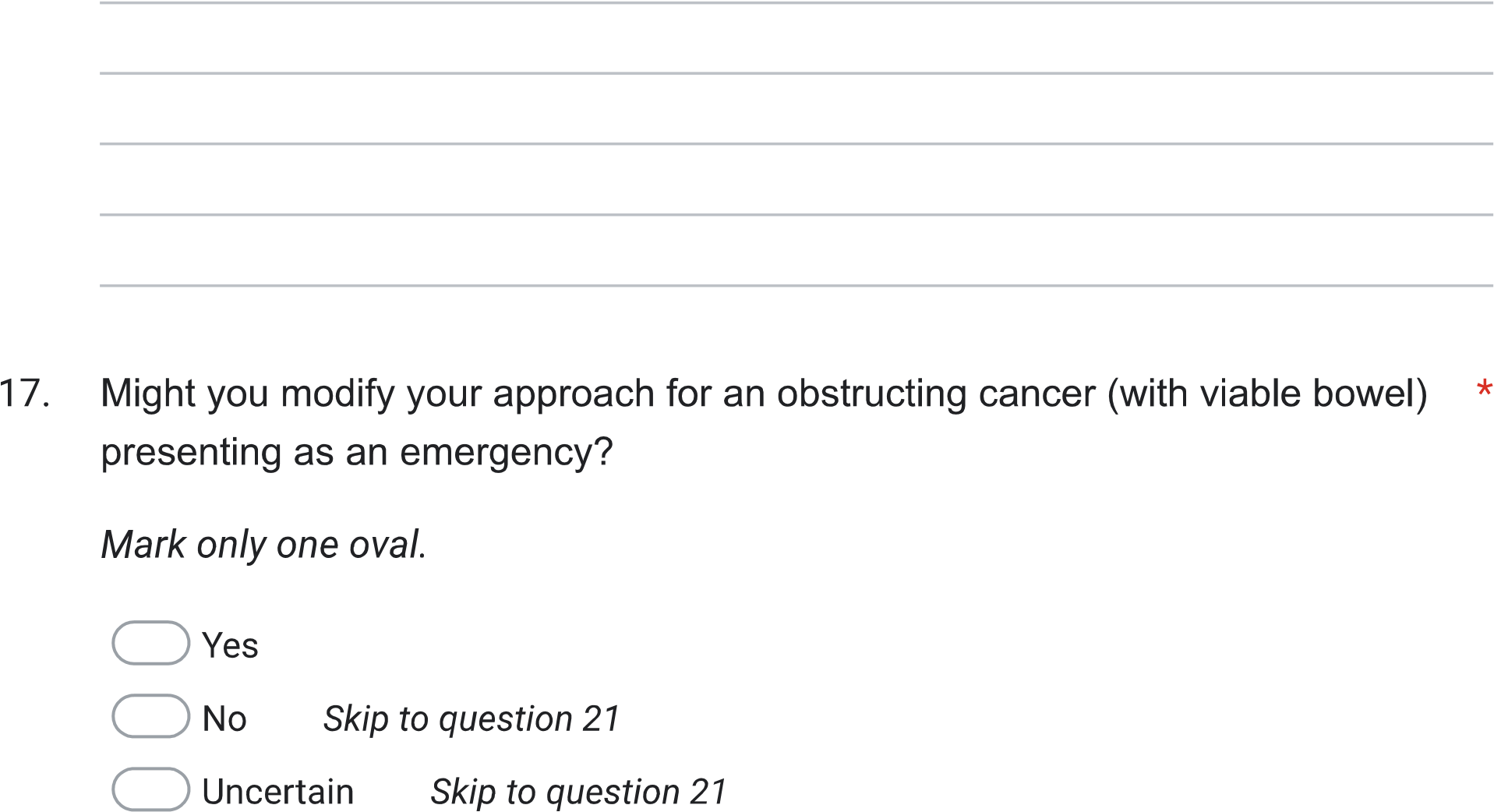

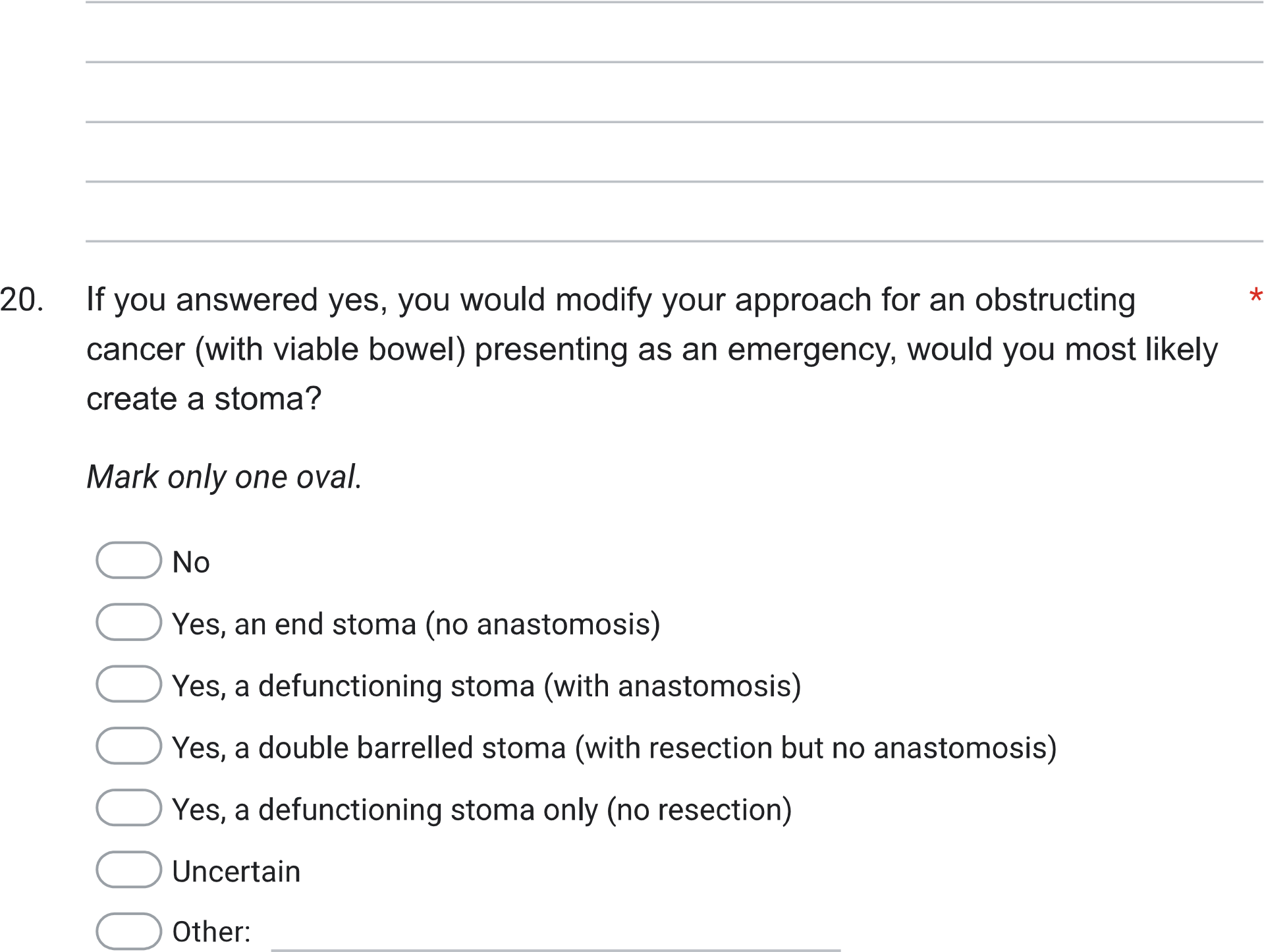

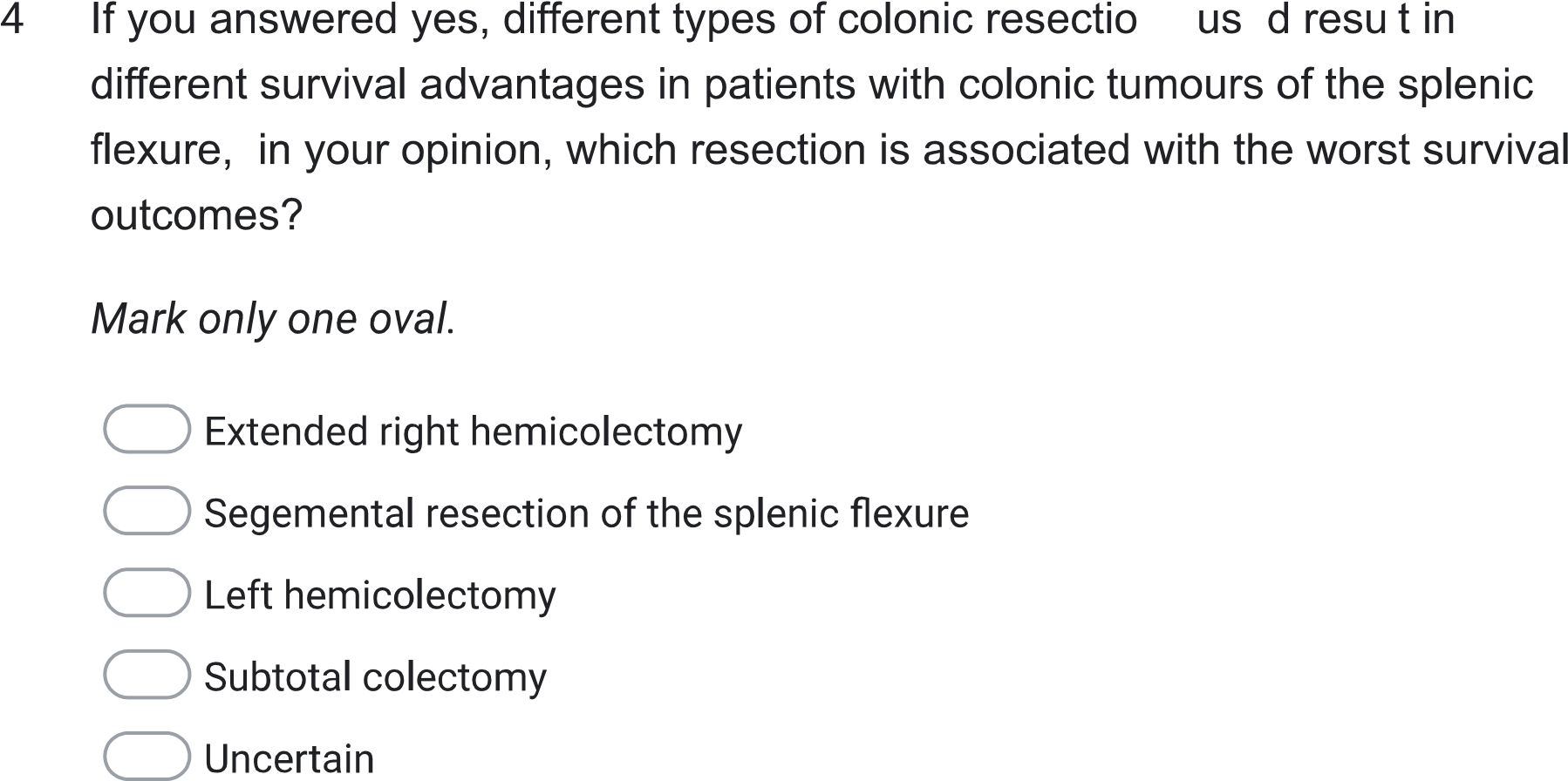


24. If you answered yes, different types of colonic resection used result in different survival advantages in patients with colonic tumours of the splenic flexure, in your opinion, which resection is associated with the worst survival outcomes?


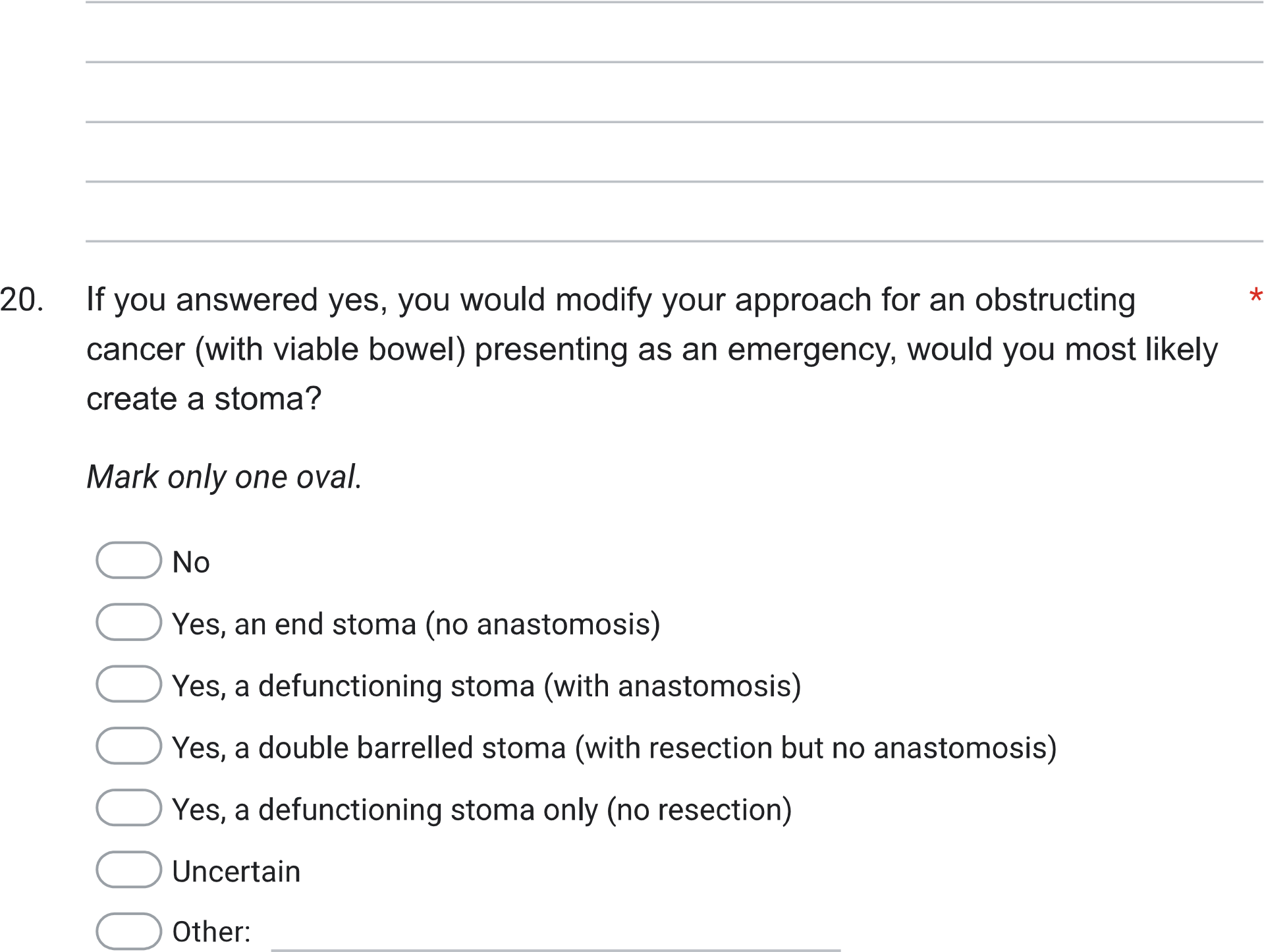


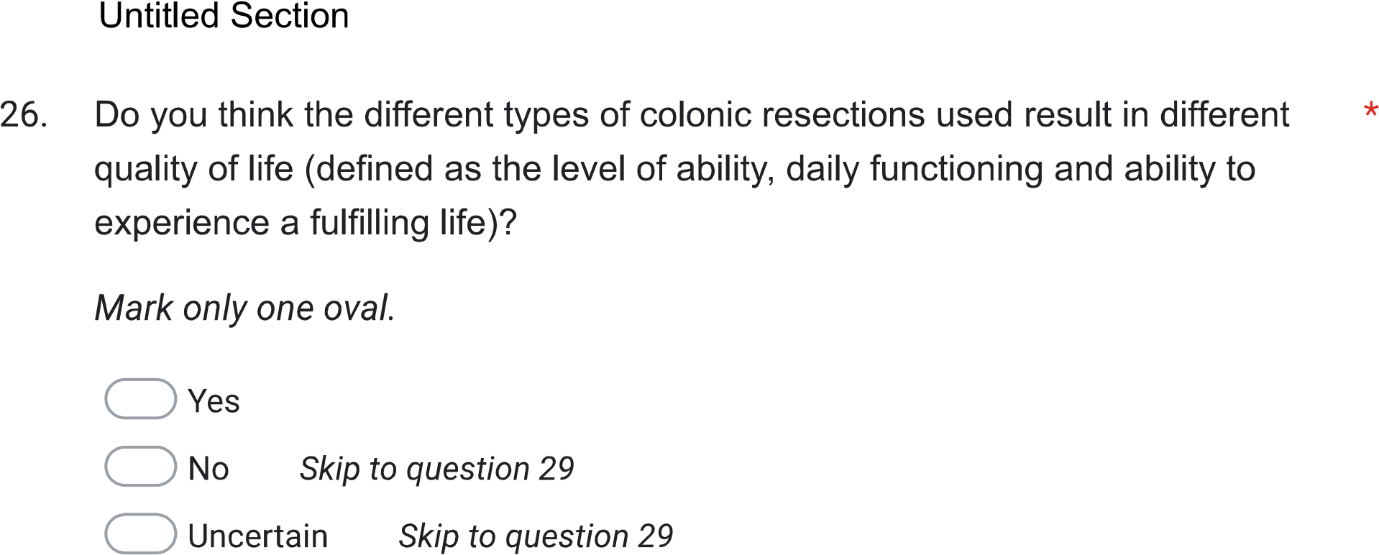


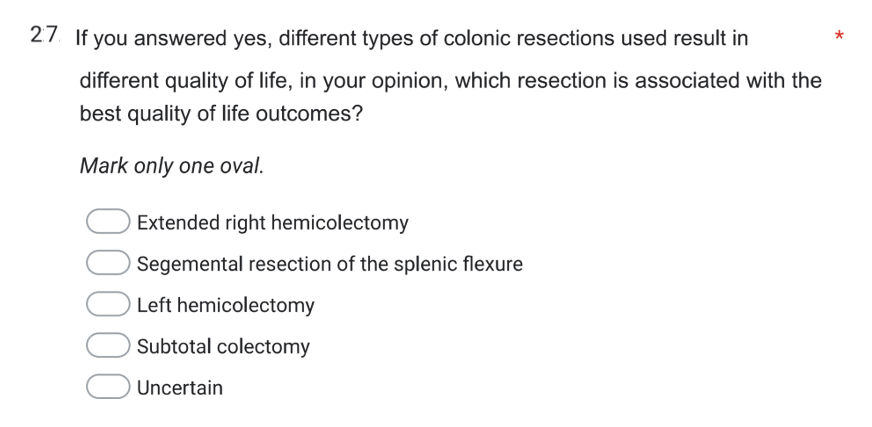

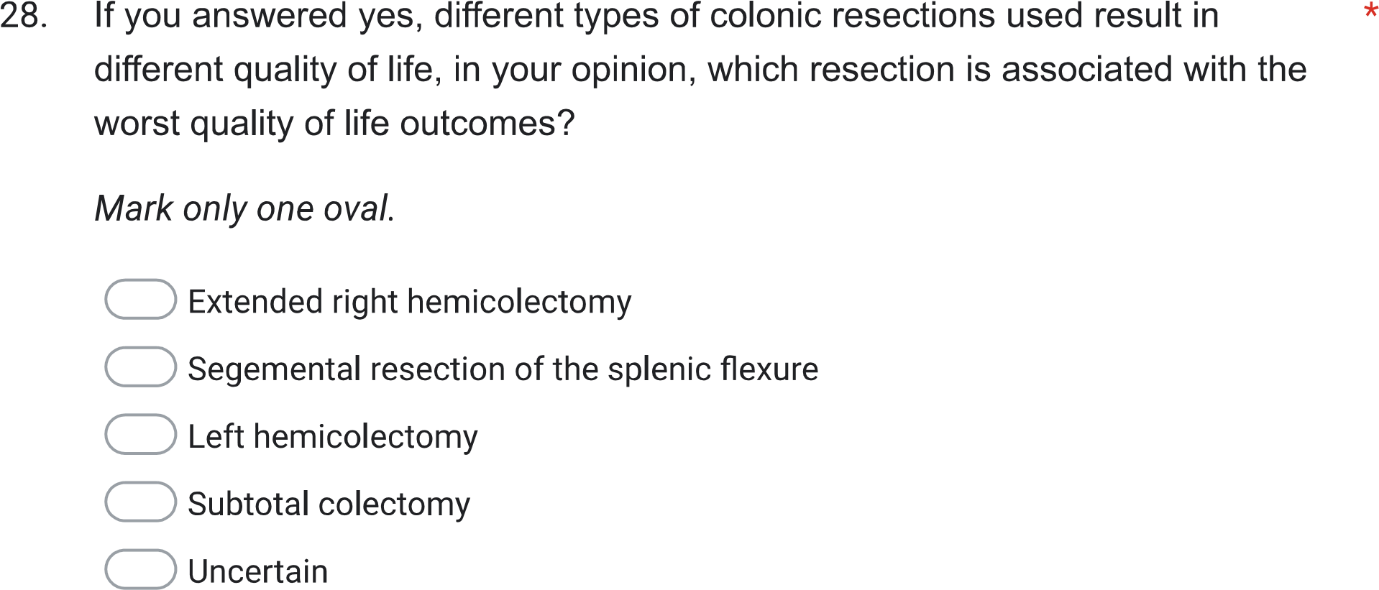


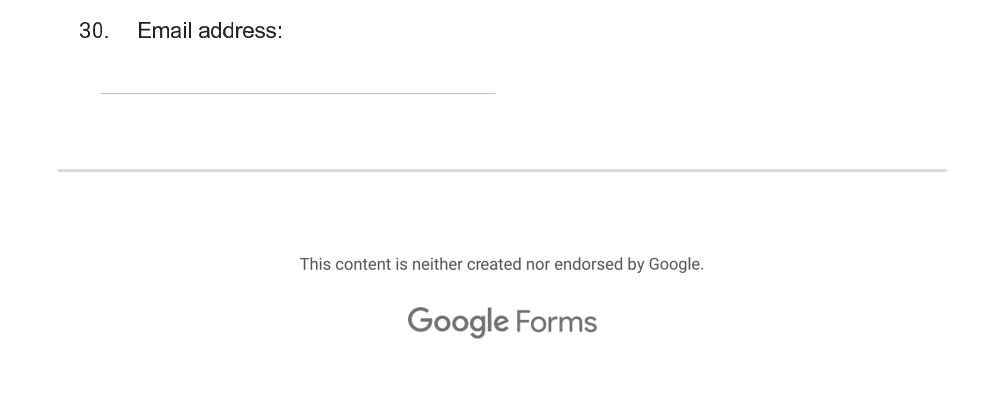

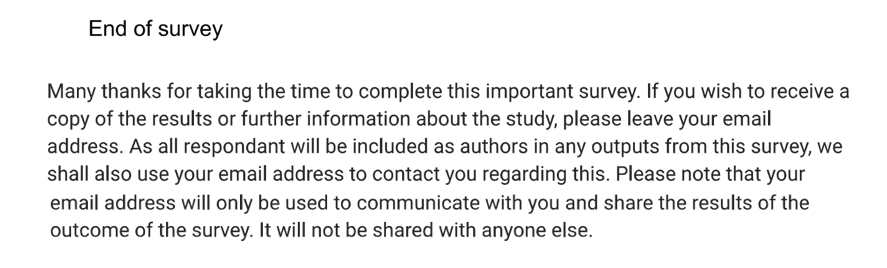

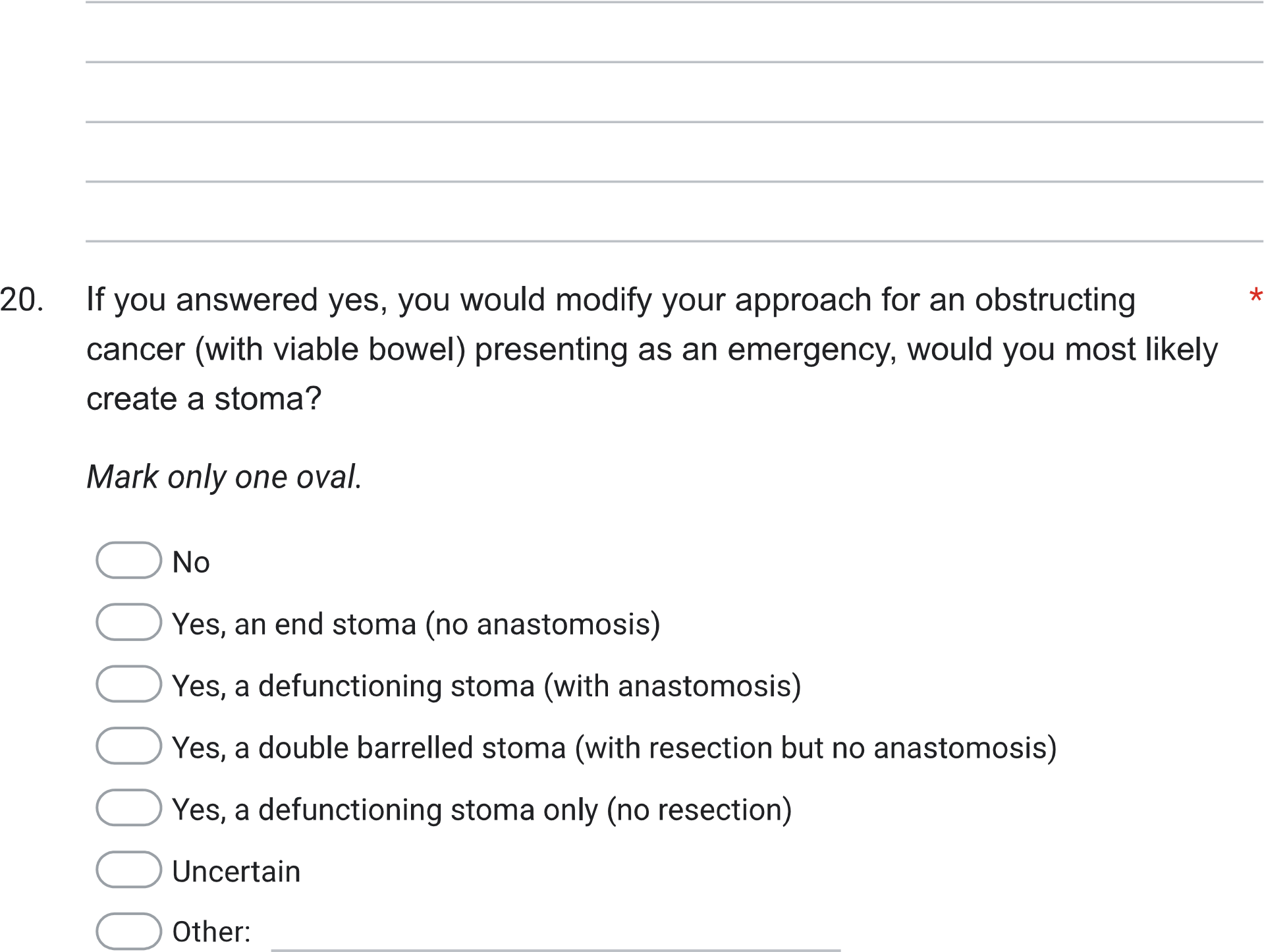


**THE INTERNATIONAL RESPONSE TO THE SURVEY**

**Table S1: The response to the survey according to country.**

| Country | Response (frequency (%)) |
| --- | --- |
| Algeria | 13 (2.3) |
| Argentina | 3 (0.5) |
| Austria | 1 (0.2) |
| Australia | 26 (4.5) |
| Azerbaijan | 5 (0.9) |
| Belgium | 2 (0.4) |
| Botswana | 1 (0.2) |
| Brazil | 21 (3.7) |
| Bulgaria | 3 (0.5) |
| Canada | 2 (0.4) |
| Chile | 1 (0.2) |
| China | 3 (0.5) |
| Denmark | 1 (0.2) |
| Republica Dominicana | 1 (0.2) |
| Egypt | 11 (1.9) |
| France | 44 (7.6) |
| Germany | 5 (0.9) |
| Ghana | 4 (0.7) |
| Greece | 10 (1.7) |
| India | 12 (2.1) |
| Indonesia | 1 (0.2) |
| Ireland | 33 (5.7) |
| Italy | 65 (11.3) |
| Israel | 1 (0.2) |
| Japan | 1 (0.2) |
| Jordan | 1 (0.2) |
| Lithuania | 7 (1.2) |
| Malaysia | 2 (0.4) |
| Morocco | 14 (2.4) |
| Mexico | 9 (1.6) |
| The Netherlands | 2 (0.4) |
| Nigeria | 30 (5.2) |
| Norway | 18 (3.1) |
| Pakistan | 1 (0.2) |
| Peru | 1 (0.2) |
| Portugal | 1 (0.2) |
| Romania | 10 (1.7) |
| Russia | 1 (0.2) |
| New Zealand | 6 (1.0) |
| Saudi Arabia | 2 (0.4) |
| Singapore | 1 (0.2) |
| South Africa | 11 (1.9) |
| Spain | 38 (6.6) |
| Sweden | 27 (4.7) |
| Turkey | 62 (10.8) |
| Tunisia | 1 (0.2) |
| Uganda | 1 (0.2) |
| UK | 49 (8.5) |
| USA | 10 (1.7) |
| Vietnam | 1 (0.2) |


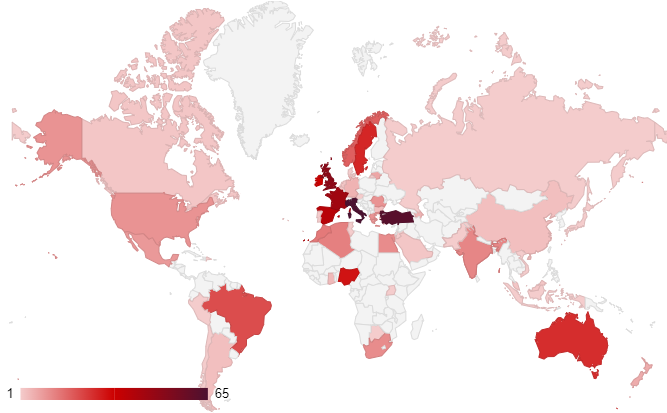


**Fig.S2: A map demonstrating the international response to the survey. The shade of the colour representing the number of respondents to the survey from each country.**

**THE STATED VASCULATURE FOR DIVISION**

**Table S2: The combination of vascular pedicles respondents stated they would divide according to the resection they stated as their preferred.**

|  | Resection (frequency (%)) | | | | | |
| --- | --- | --- | --- | --- | --- | --- |
| Combination of pedicles divided  (frequency (%)) | ERH  (126 (21.9)) | LH  (203 (35.2)) | SEG  (167 (29.0)) | STC  (38 (6.6)) | Other  (35 (6.1)) | Uncertain  (7 (1.2)) |
| ICA, RMCA (1 (0.2)) | 1 (0.8) | 0 | 0 | 0 | 0 | 0 |
| ICA, RMCA, IMA (1 (0.2)) | 1 (0.8) | 0 | 0 | 0 | 0 | 0 |
| ICA, MCA (34 (5.9)) | 33 (26.2) | 0 | 0 | 1 (2.6) | 0 | 0 |
| ICA, MCA, RMCA (1 (0.2)) | 1 (0.8) | 0 | 0 | 0 | 0 | 0 |
| ICA, MCA, RMCA, LMCA (22 (3.8)) | 20 (15.9) | 0 | 0 | 2 (5.3) | 0 | 0 |
| ICA, MCA, RMCA, LMCA, LCA (30 (5.2)) | 21 (16.7) | 0 | 0 | 7 (18.4) | 2 (5.7) | 0 |
| ICA, MCA, RMCA, LMCA, LCA, IMA (3 (0.5)) | 1 (0.8) | 0 | 0 | 2 (5.3) | 0 | 0 |
| ICA, MCA, RMCA, LMCA, LCA, Uncertain  (1 (0.2) | 1 (0.8) | 0 | 0 | 0 | 0 | 0 |
| ICA, MCA, RMCA, LMCA, IMA (1 (0.2)) | 0 | 0 | 0 | 0 | 0 | 1 (14.3) |
| ICA, MCA, LMCA (1 (0.2)) | 1 (0.8) | 0 | 0 | 0 | 0 | 0 |
| ICA, MCA, LMCA, LCA (1 (0.2)) | 1 (0.8) | 0 | 0 | 0 | 0 | 0 |
| ICA, MCA, LMCA, LCA, Other (1 (0.2)) | 0 | 0 | 0 | 0 | 1 (2.9) | 0 |
| ICA, MCA, LCA (26 (4.5)) | 15 (11.9) | 0 | 0 | 9 (23.7) | 2 (5.7) | 0 |
| ICA, MCA, LCA, Other (1 (0.2)) | 1 (0.8) | 0 | 0 | 0 | 0 | 0 |
| ICA, MCA, IMA (1 (0.2)) | 0 | 0 | 0 | 1 (2.6) | 0 | 0 |
| ICA, MCA, Other (5 (0.9)) | 4 (3.2) | 0 | 0 | 0 | 1 (2.9) | 0 |
| RMCA (3 (0.5)) | 1 (0.8) | 0 | 2 (1.2) | 0 | 0 | 0 |
| RMCA, LMCA (3 (0.5)) | 1 (0.8) | 1 (0.5) | 1 (0.6) | 0 | 0 | 0 |
| RMCA, LMCA, LCA (1 (0.2)) | 0 | 0 | 0 | 0 | 1 (2.9) | 0 |
| RMCA, LMCA, IMA (1 (0.2)) | 0 | 1 (0.5) | 0 | 0 | 0 | 0 |
| RMCA, LCA (5 (0.9)) | 0 | 2 (1.0) | 2 (1.2) | 0 | 1 (2.9) | 0 |
| MCA (8 (1.4)) | 5 (4.0) | 1 (0.5) | 2 (1.2) | 0 | 0 | 0 |
| MCA, RMCA (1 (0.2)) | 0 | 1 (0.5) | 0 | 0 | 0 | 0 |
| MCA, RMCA, LMCA (2 (0.4)) | 0 | 0 | 2 (1.2) | 0 | 0 | 0 |
| MCA, RMCA, LMCA, LCA (1 (0.2)) | 0 | 0 | 1 (0.6) | 0 | 0 | 0 |
| MCA, LMCA 2 (0.4)) | 0 | 0 | 1 (0.6) | 1 (2.6) | 0 | 0 |
| MCA, LMCA, LCA (4 (0.7)) | 0 | 1 (0.5) | 2 (1.2) | 1 (2.6) | 0 | 0 |
| MCA, LMCA, LCA, IMA (2 (0.4)) | 0 | 2 (1.0) | 0 | 0 | 0 | 0 |
| MCA, LCA (18 (3.1)) | 4 (3.2) | 7 (3.5) | 3 (1.8) | 4 (10.5) | 0 | 0 |
| MCA, LCA, IMA (1 (0.2)) | 0 | 1 (0.5) | 0 | 0 | 0 | 0 |
| MCA, LCA, Uncertain (1 (0.2)) | 0 | 0 | 1 (0.6) | 0 | 0 | 0 |
| MCA, IMA (5 (0.9)) | 0 | 4 (2.0) | 0 | 0 | 1 (2.9) | 0 |
| LMCA (14 (2.4)) | 1 (0.8) | 5 (2.5) | 3 (1.8) | 3 (7.9) | 2 (5.7) | 0 |
| LMCA, LCA (230 (39.9)) | 10 (7.9) | 93 (45.8) | 113 (67.7) | 5 (13.2) | 7 (20.0) | 2 (28.6) |
| LMCA, LCA, IMA (34 (5.9)) | 0 | 29 (14.3) | 3 (1.8) | 0 | 2 (5.7) | 0 |
| LMCA, LCA, Other (3 (0.5)) | 0 | 1 (0.5) | 2 (1.2) | 0 | 0 | 0 |
| LMCA, IMA (20 (3.5)) | 2 (1.6) | 14 (6.9) | 0 | 1 (2.6) | 3 (8.6) | 0 |
| LMCA, Other (1 (0.2)) | 0 | 0 | 1 (0.6) | 0 | 0 | 0 |
| LMCA, Uncertain (1 (0.2)) | 0 | 1 (0.5) | 0 | 0 | 0 | 0 |
| LCA (46 (8.0)) | 1 (0.8) | 20 (9.9) | 23 (13.8) | 0 | 0 | 2 (28.6) |
| LCA, IMA (5 (0.9)) | 0 | 3 (1.5) | 1 (0.6) | 0 | 1 (2.9) | 0 |
| LCA, Uncertain (1 (0.20)) | 0 | 1 (0.5) | 0 | 0 | 0 | 0 |
| IMA (16 (2.8)) | 0 | 15 (7.4) | 1 (0.6) | 0 | 0 | 0 |
| Other (13 (2.3)) | 0 | 0 | 2 (1.2) | 0 | 11 (31.4) | 0 |
| Uncertain (4 (0.7)) | 0 | 0 | 1 (0.6) | 1 (2.6) | 0 | 2 (28.6) |

ERH: extended right hemicolectomy; LH: left hemicolectomy; SEG: segmental resection; STC: subtotal colectomy; ICA: ileocolic artery; MCA: middle colic artery; RMCA: right branch of the middle colic artery; LMCA: left branch of middle colic artery; LCA: left colic artery; IMA: inferior mesenteric artery.

Other arteries that were proposed for division included the left ascending branch of the left colic artery, the sigmoid arteries, the right colic artery and the artery of Moskowitz.

**MODIFICATIONS TO OPERATIVE APPROACH IN A PATIENT DEEMED TO BE OF ‘HIGH RISK’**

**Table S3: Association of whether respondents would modify their preferred resection in a patient deemed to be ‘high risk’ and the resection type the respondents originally stated they preferred for splenic flexure cancers.**

|  | Resection (frequency (%)) | | | | | |
| --- | --- | --- | --- | --- | --- | --- |
| Modify for emergency (frequency (%)) | ERH  (126 (21.9)) | LH  (203 (35.2)) | SEG  (167 (29.0)) | STC  (38 (6.6)) | Other  (35 (6.1)) | Uncertain  (7 (1.2)) |
| Yes (318 (55.2) | 82 (65.1) | 135 (66.5) | 54 (32.3) | 23 (60.5) | 20 (57.1) | 4 (57.1) |
| No (222 (38.5)) | 35 (27.8) | 58 (28.6) | 101 (60.5) | 13 (34.2) | 14 (40.0) | 1 (14.3) |
| Uncertain (36 (6.3)) | 9 (7.1) | 10 (4.9) | 12 (7.2) | 2 (5.3) | 1 (2.9) | 2 (28.6) |

ERH: extended right hemicolectomy; LH: left hemicolectomy; SEG: segmental resection; STC: subtotal colectomy.

**Table S4: Association of which resection respondents would modify their operative approach to if they stated that they would modify their operative approach in a patient deemed ‘high risk’ and the resection type the respondents originally stated they preferred for splenic flexure cancers.**

|  | Resection (frequency (%)) | | | | | | |
| --- | --- | --- | --- | --- | --- | --- | --- |
| If yes, which modification (frequency (%)) | ERH  (82 (25.8)) | LH  (135 (42.5)) | SEG  (54 (17.0)) | STC  (23 (7.2)) | Other  (20 (6.3)) | Uncertain  (4 (1.3)) |  |
| ERH (24 (7.6)) | 6 (7.3) | 8 (5.9) | 8 (14.8) | 1 (4.4) | 1 (5.0) | 0 |  |
| SEG (204 (64.2)) | 56 (68.3) | 91 (67.4) | 34 (63.0) | 9 (39.1) | 13 (65.0) | 1 (25.0) |  |
| LH (48 (6.9)) | 15 (18.3) | 18 (13.3) | 5 (9.3) | 0 | 4 (20.0) | 0 |  |
| STC (22 (6.9)) | 1 (1.2) | 11 (8.2) | 2 (3.7) | 1 (25.0) | 1 (5.0) | 1 (25.0) |  |
| Other (13 (4.1))* | 4 (30.8) | 5 (3.7) | 2 (5.6) | 0 | 0 | 0 |  |
| Uncertain (7 (2.2)) | 0 | 2 (1.5) | 2 (3.7) | 2 (50.0) | 1 (5.0) | 2 (50.0) |  |

ERH: extended right hemicolectomy; LH: left hemicolectomy; SEG: segmental resection; STC: subtotal colectomy.

*Including performing a low ligation of the IMA, stenting the tumour to allow for patient optimisation prior to performing a definitive resection, performing a resection and creating a stoma, performing a defunctioning stoma to facilitate chemotherapy and that they were most likely to employ and open approach.


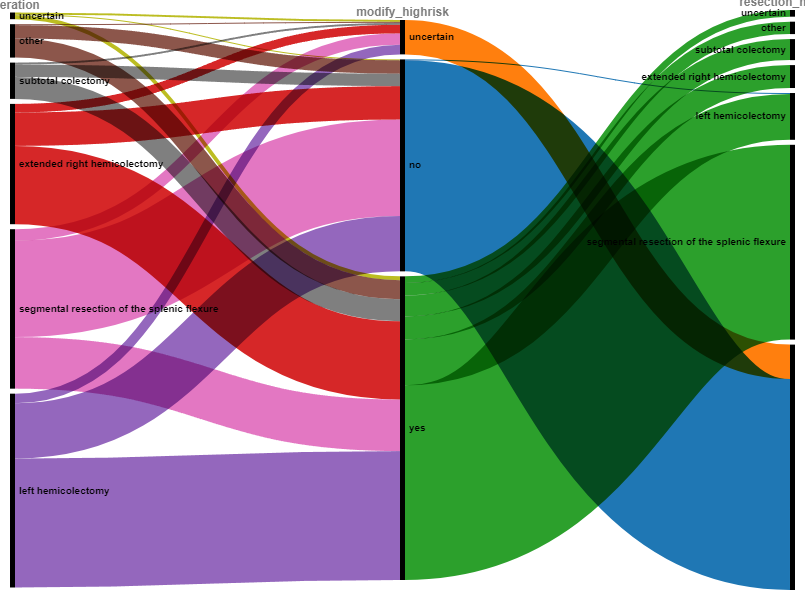


**Fig.S3: An alluvial diagram demonstrating proportional correlations between the original resection type respondents stated they preferred for splenic flexure cancers (on the left), whether they would modify this in a patient deemed ‘high risk’ (in the centre) and what resection they would modify to in a patient deemed ‘high risk’ (on the right).**

**MODIFICATIONS TO OPERATIVE APPROACH IN A PATIENT PRESENTING AS AN EMERGENCY WITH BOWEL OBSTRUCTION**

**Table S5: Association of whether respondents would modify their preferred resection in a patient presenting as an emergency with obstructed bowel and the resection type the respondents originally stated they preferred for splenic flexure cancers.**

|  | Resection (frequency (%)) | | | | | |
| --- | --- | --- | --- | --- | --- | --- |
| Modify for emergency (frequency (%)) | ERH  (126 (21.9)) | LH  (203 (35.2)) | SEG  (167 (29.0)) | STC  (38 (6.6)) | Other  (35 (6.1)) | Uncertain  (7 (1.2)) |
| Yes (375 (65.2)) | 66 (52.4) | 147 (72.4) | 123 (74.1) | 20 (52.6) | 15 (42.9) | 4 (57.1) |
| No (175 (30.4)) | 57 (45.2) | 50 (24.6) | 33 (19.9) | 18 (47.4) | 16 (45.7) | 1 (14.3) |
| Uncertain (25 (4.4)) | 3 (2.4) | 6 (3.0) | 10 (6.0) | 0 | 4 (11.4) | 2 (28.6) |

ERH: extended right hemicolectomy; LH: left hemicolectomy; SEG: segmental resection; STC: subtotal colectomy.

**Table S6: Association of which resection respondents would modify their operative approach to if they stated that they would modify their operative approach in a patient presenting as an emergency with obstructed bowel and the resection type the respondents originally stated they preferred for splenic flexure cancers.**

|  | Resection (frequency (%)) | | | | | |
| --- | --- | --- | --- | --- | --- | --- |
| If yes, which modification  (frequency (%)) | ERH  (66 (17.6)) | LH  (147 (39.2)) | SEG  (123 (32.8)) | STC  (20 (5.3)) | Other  (15 (4.0)) | Uncertain  (4 (1.1)) |
| ERH (85 (22.7)) | 17 (25.8) | 22 (15.0) | 39 (31.7) | 2 (10.0) | 4 (26.7) | 1 (25.0) |
| SEG (35 (9.3)) | 10 (15.2) | 14 (9.5) | 8 (6.5) | 2 (10.0) | 1 (6.7) | 0 |
| LH (29 (7.7)) | 3 (4.6) | 18 (12.2) | 8 (6.5) | 0 | 0 | 0 |
| STC (71 (18.9)) | 6 (9.1) | 32 (21.8) | 18 (14.6) | 11 (55.0) | 3 (20.0) | 1 (25.0) |
| Colonic stent (53 (14.1)) | 14 (21.2) | 17 (11.6) | 17 (13.8) | 2 (10.0) | 1 (6.7) | 2 (50.0) |
| Other (93 (24.8))* | 15 (22.7) | 42 (28.6) | 31 (25.2) | 2 (10.0) | 3 (20.0) | 0 |
| Uncertain (9 (2.4)) | 1 (1.5) | 2 (1.4) | 2 (1.6) | 1 (5.0) | 3 (20.0) | 0 |

ERH: extended right hemicolectomy; LH: left hemicolectomy; SEG: segmental resection; STC: subtotal colectomy.

*Most expressed that they would form a stoma. Whilst some stated the stoma would be to avoid an anastomosis, with or without consideration of restoration of bowel continuity in the future, many others stated that they would use it as a bridge to perform a two-stage procedure. In this later scenario, having performed a diverting stoma, respondents stated they would proceed to stage and optimise the patient, including oncologic staging, oncogeriatric and nutritional evaluation and rehabilitation, in order to them perform a definitive resection. A few even stated the timeline to the definitive procedure to be over the next 8 to 10 days. Some respondents stated that they would perform a resection open rather than laparoscopically in the emergency setting and other techniques mentioned included on table lavage during an emergency resection to facilitate an anastomosis.


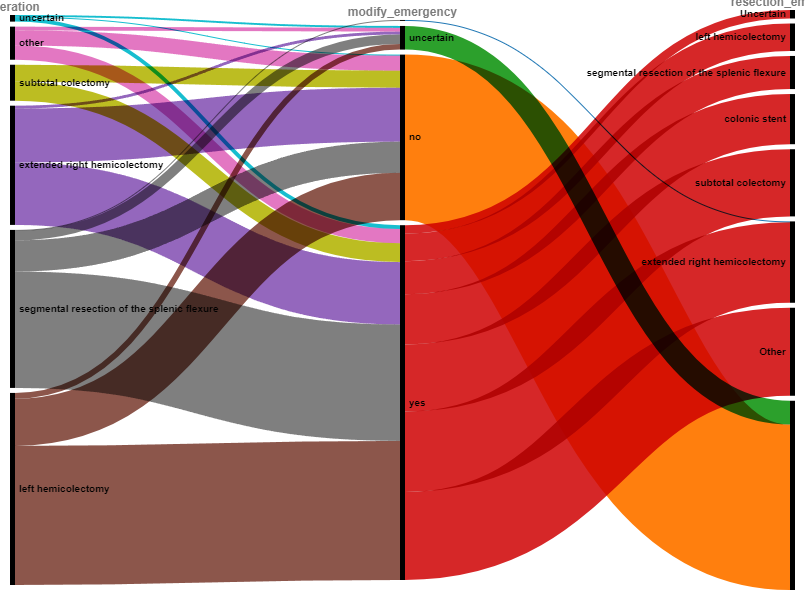
**Fig.S4: An alluvial diagram demonstrating proportional correlations between the original resection type respondents stated they preferred for splenic flexure cancers (on the left), whether they would modify this in a patient presenting as an emergency with obstructed bowel and what resection they would modify to in a patient presenting as an emergency with obstructed bowel (on the right).**

**Question: if you answered yes, you would modify your approach for an obstructing cancer (with viable bowel) presenting as an emergency, would you most likely create a stoma?**

The majority 464 (70%) stated yes, they would most likely create a stoma and of these, 80 (21%) would create an end stoma without a bowel anastomosis; 40 (11%) would create a defunctioning stoma with a bowel anastomosis, 40 (11%) would create a double-barrelled stoma having performed a resection but without an anastomosis and 69 (18%) would perform a defuncitoning stoma only, without a resection. 21 (6%) were uncertain and 16 (4%) stated other including once more reinforcing the use of a defunctioning stoma with a second stage procedure of a definitive resection and restoration of bowel continuity, whilst a few stated they would use a colonic stent as a bridge to definitive surgery. Many of the comments referred to the decisions being dependent on the patients’ clinical condition, the quality of the bowel, patient morbidity as well as anatomy. One person stated that they would use a colonic stent as a bridge and then perform a laparoscopic left-sided complete mesocolic excision. It was reinforced that the strategy would be dependent upon and tailored to the patient.
